# Easy-MODA: Simplifying standardised registration of scientific simulation workflows through MODA template guidelines powered by the Enalos Cloud Platform

**DOI:** 10.1016/j.csbj.2024.10.018

**Published:** 2024-10-18

**Authors:** Panagiotis D. Kolokathis, Nikolaos K. Sidiropoulos, Dimitrios Zouraris, Dimitra-Danai Varsou, Dimitris G. Mintis, Andreas Tsoumanis, Francesco Dondero, Thomas E. Exner, Haralambos Sarimveis, Evgenia Chaideftou, Martin Paparella, Fotini Nikiforou, Achilleas Karakoltzidis, Spyros Karakitsios, Dimosthenis Sarigiannis, Jesper Friis, Gerhard Goldbeck, David A. Winkler, Willie Peijnenburg, Angela Serra, Dario Greco, Georgia Melagraki, Iseult Lynch, Antreas Afantitis

**Affiliations:** a NovaMechanics MIKE, Piraeus 18545, Greece; b Entelos Institute, Larnaca 6059, Cyprus; c NovaMechanics Ltd, Nicosia 1070, Cyprus; d Department of Science and Technological Innovation, Università del Piemonte Orientale, 15121 Alessandria, Italy; e SevenPastNine GmbH, Rebacker 68, 79650 Schopfheim, Germany; f School of Chemical Engineering, National Technical University of Athens, 15780 Zografou, Greece; g Department of Medical Biochemistry, Medical University of Innsbruck, Innsbruck, Austria; h Aristotle University of Thessaloniki, Department of Chemical Engineering, Environmental Engineering Laboratory, University Campus, Thessaloniki 54124, Greece; i Department of Materials and Nanotechnology, SINTEF Industry, Trondheim N-7465, Norway; j European Materials Modelling Council, Brussels 1050, Belgium; k Goldbeck Consulting Limited, Cambridge CB4 0WS, UK; l La Trobe Institute for Molecular Science, La Trobe University, Bundoora, Australia; m Monash Institute of Pharmaceutical Sciences, Monash University, Parkville, Australia; n School of Pharmacy, University of Nottingham, Nottingham, UK; o Institute of Environmental Sciences (CML), Leiden University, Leiden 2300 RA, The Netherlands; p Centre for Safety of Substances and Products, National Institute of Public Health and the Environment (RIVM), Bilthoven 3720, The Netherlands; q FHAIVE, Faculty of Medicine and Health Technology, Tampere University, Finland; r Division of Physical Sciences and Applications, Hellenic Military Academy, Vari 16672, Greece; s School of Geography, Earth and Environmental Sciences, University of Birmingham, Birmingham B15 2TT, UK

**Keywords:** Easy-MODA, MODA, Scientific simulation workflows, QMRF, CEN Workshop Agreement (CWA 17284:2018)

## Abstract

Modelling Data (MODA) reporting guidelines have been proposed for common terminology and for recording metadata for physics-based materials modelling and simulations in a CEN Workshop Agreement (CWA 17284:2018). Their purpose is similar to that of the Quantitative Structure-Activity Relationship (QSAR) model report form (QMRF) that aims to increase industry and regulatory confidence in QSAR models, but for a wider range of model types. Recently, the WorldFAIR project’s nanomaterials case study suggested that both QMRF and MODA templates are an important means to enhance compliance of nanoinformatics models, and their underpinning datasets, with the FAIR principles (Findable, Accessible, Interoperable, Reusable). Despite the advances in computational modelling of materials properties and phenomena, regulatory uptake of predictive models has been slow. This is, in part, due to concerns about lack of validation of complex models and lack of documentation of scientific simulations. The models are often complex, output can be hardware- and software-dependent, and there is a lack of shared standards. Despite advocating for standardised and transparent documentation of simulation protocols through its templates, the MODA guidelines are rarely used in practice by modellers because of a lack of tools for automating their creation, sharing, and storage. They also suffer from a paucity of user guidance on their use to document different types of models and systems. Such tools exist for the more well-established QMRF and have aided widespread implementation of QMRFs. To address this gap, a simplified procedure and online tool, Easy-MODA, has been developed to guide users through MODA creation for physics-based and data-based models, and their various combinations. Easy-MODA is available as a web-tool on the Enalos Cloud Platform (https://www.enaloscloud.novamechanics.com/insight/moda/). The tool streamlines the creation of detailed MODA documentation, even for complex multi-model workflows, and facilitates the registration of MODA workflows and documentation in a database, thereby increasing their Findability and thus Re-usability. This enhances communication, interoperability, and reproducibility in multiscale materials modelling and improves trust in the models through improved documentation. The use of the Easy-MODA tool is exemplified by a case study for nanotoxicity evaluation, involving interlinked models and data transformation, to demonstrate the effectiveness of the tool in integrating complex computational methodologies and its significant role in improving the FAIRness of scientific simulations.

## Graphical Abstract

**Figure:**
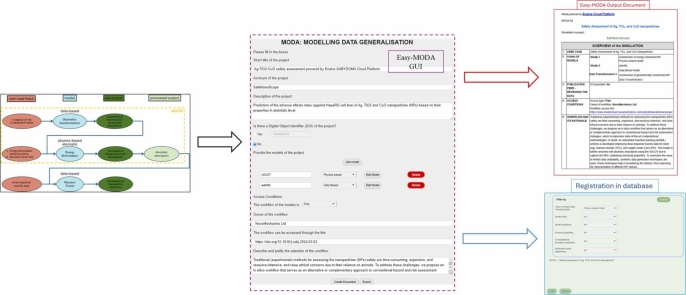

## 1 Introduction

The reproducibility of simulations across different scientific domains faces significant challenges. These include model complexity, strict accuracy requirements, the variability of hardware and software environments, and the lack of standardisation of simulation documentation and execution. For instance, specialised software and hardware can lead to differing outcomes due to differing default software options and hardware settings such as memory, and central and graphics processing units (CPU / GPU) usage, which depend on the user's infrastructure and input. These sources of variability are commonly encountered in simulations requiring stochastic methods and Monte Carlo methods for complex optimization problems and numerical integration [1]. For example, inconsistencies between user groups result from working with different atomistic force fields (i.e., a set of functions and parameters that describe the forces exerted among the atoms during atomistic simulations) or materials relations (e.g., a set of different functions and parameters that describe the material’s properties used during continuum model simulations such as density, viscosity, elastic modulus, etc.) and simulation programs (e.g., LAMMPS, GROMACS, OPENFOAM, CP2K etc.). This underscores the urgent need for standardised documentation of simulations to clarify model inputs and outputs, make the models more reproducible by others, and thereby increase model interoperability and reusability [2], [3].

Simulation reporting challenges are not only technical but also methodological, with a notable lack of consensus on definitions and standards for reproducibility. This leads to variations in practices across different scientific domains [4]. For example, in computational neuroscience, small variations in numerical implementation across simulation packages can significantly affect results, necessitating the adoption of standards for model specification [5]. Similarly, models developed by the same team with different software can face major reproducibility issues, indicating a need for multistage validation and facilitation of replication studies through source code publication [6]. Moreover, insufficient detail in model definitions, including parameters and equations, hinders reproducibility and interoperability. This highlights the need for declarative model descriptions (or metadata about the models) through comprehensive model documentation [7]. Additionally, technical issues such as software bugs, complications with floating point roundoff, and evolving computer systems and architectures underscore the critical need for reuse of published software configurations or justification for adoption of new ones. Consequently, providing documentation according to a standard, such as MODA, is urgently needed. Providing detailed specifications for computer experiments will help to ensure reproducibility without necessarily reusing the same source code [8].

These multifaceted challenges underscore the importance of adopting best practices in software development, detailed documentation of computing environments, and establishing community-wide standards for model and software reproducibility. To address these simulation reproducibility challenges across scientific domains, the Modelling Data (MODA) framework emerged from the European Commission's Horizon 2020 Framework Programme under the auspices of the European Materials Modelling Council (EMMC). MODA addresses the critical need for standardisation of materials models and enhanced documentation of modelling activities, offering a structured approach to detail key aspects of modelling work. The documentation (metadata) covers all aspects of the modelling including any physics equations, materials relations, solvers utilised, and both pre- and post-processing techniques, along with the workflow of models in the case of model integration. It was formalised by a CEN Workshop Agreement (CWA 17284 “Materials modelling - terminology, classification and metadata”) [9]. The goal of the MODA is to systematise the reporting of physics-based models and extend this to reporting of data-based models, and modelling workflows that integrate both physics-based and other models such as data-driven/data-based and empirical models. In the following they will be referred to simply by the term data-based. For quantitative structure-activity relationship (QSAR) models, a subcategory of data-based models, the standardised QSAR model report form (QMRF) is often used [10]. This template has been developed and refined over several years and is required to accompany all QSAR models proposed for use in regulatory risk assessment [11]. QMRF is thus a mature reporting standard that has stimulated development of multiple tools for generation of QMRFs that support user model documentation. Easy-MODA, presented here, represents the first such tool for automating the generation of MODAs.

The ever increasing number of multiscale physics-based simulations [12], [13], [14], machine learning models (data-based models trained on large datasets that identify patterns e.g., QSAR models), and Integrated Approaches to Testing and Assessment (IATA) that can combine the two types of modelling into a single, integrated multi-model workflow [15] demands a standard way of representing their workflows. To the end, MODA supports the development of increasingly complex workflows based on prior versions created by industrial end-users, software developers, and theoreticians. For example, many of the tools deployed on the Enalos Cloud Platform (https://www.enaloscloud.novamechanics.com/all.html) have detailed MODAs and/or QMRFs that depend on the type of model as a means to increase the usability of, and confidence in, the models. The models can be integrated through Application Programming Interface (API) calls to tackle specific problems. MODA aids these integrations, aligns with the EU's Data Act, and aims to make the models and their underpinning equations or data FAIR (Findable, Accessible, Interoperable, Reusable) in accordance with the FAIR data principles [16]. A recent evaluation of the steps required to make materials and nanoinformatics models and software FAIR, performed via the Nanomaterials Case Study of the WorldFAIR project and reported in Deliverable D4.2 on FAIRification of models and software [17], identified MODA and QMRFs as FAIR Enabling Resources providing metadata schema and potentially linking metadata and data. By standardising simulation documentation and improving simulation data management, MODA can facilitate model interoperability (the I of FAIR) and reusability (the R of FAIR) by making complex modelling processes more approachable and understandable for various stakeholders. By establishing a common terminology (a structured vocabulary or ontology) and framework, MODA effectively bridges the communication gap between industrial end-users, software developers, academia, and theoreticians. MODA also allows monitoring of the propagation of errors produced by different models in a workflow, providing further understanding of the reliability of workflow results [18]. However, MODA is rarely applied in practice, mainly due to the lack of tools for automation of their creation, storing and sharing them, and a lack of the ecosystem of services around it that QMRF benefits from. To bridge this gap, we present here an automated tool to support users in completing MODA templates using the MODA guidelines. It provides pre-filling options based on user selections to simplify the procedure and promote much greater usage of MODA templates, while simultaneously reducing the risk of errors associated with free-text entries.

This paper describes Easy-MODA, a free-to-use web tool hosted on the Enalos Cloud Platform. Easy-MODA aims to significantly increase the MODA framework's accessibility and user engagement by automating and simplifying the MODA documentation process through comprehensive online forms and integrated guidance. One area of concern for MODA documentation is the wide range of options available for the MODA template fields [19], which are often interdependent, having many possible combinations. By pre-identifying these interdependencies, Easy-MODA automatically generates compatible options for each template field based on prior choices made by the user, and guides the user on how to fill the template quickly and accurately. Furthermore, where models are combined, the resulting MODA are also more complex. This is increasingly the case for materials models that span multiple scales and nanoinformatics models that predict nanomaterials fate, impacts (toxicity) and overall risk via Integrated approaches to Testing and Assessment (IATA). Easy-MODA supports users adding multiple models to a project and overcomes the barrier of increasing complexity of the manual operations required for the MODA template using intelligent automation. While the original CEN Workshop Agreement (CWA 17284:2018) focused entirely on physics-based models, use of MODA in the EMMC and related projects performed after the publication of the CWA 17284:2018 showed that the MODA concept can also be applied to data-based modelling (see https://emmc.eu/moda/ and Ref. 19). To ease the use for data-based models, Easy-MODA matches the fields of the QMRF with the corresponding fields of MODA.

To exemplify the benefits of the Easy-MODA web tool, we applied it to the modelling methodology developed by Varsou et al. [20], which leverages advanced computational techniques to assess nanomaterial toxicity. This approach resonates with MODA's mission to standardise the documentation of materials modelling activities, especially those involving different computational methods (e.g., physics-based and data-based methods). The study by Varsou et al. [20] presents an automated machine learning (autoML) scheme that employs dose-response toxicity data for silver (Ag), titanium dioxide (TiO_2_), and copper oxide (CuO) nanoparticles (NPs) and computationally derived atomistic descriptors to model the NPs’ underlying structural properties (physics-based modelling). Using the Varsou et al. [20] study, we demonstrate the EasyMODA platform's capacity to systematise sophisticated, data-driven research, and combine it with physics-based predictions, enhancing reproducibility, reducing uncertainty and encouraging data sharing and model re-use within the scientific community. While workflow optimization is not a primary focus of Easy-MODA, the tool’s structured approach to documentation can inherently help researchers review their workflows for inefficiencies.

## 2 Description of Easy-MODA’s Graphical User Interface (GUI), its background logic and its capabilities

**Fig. 1:**
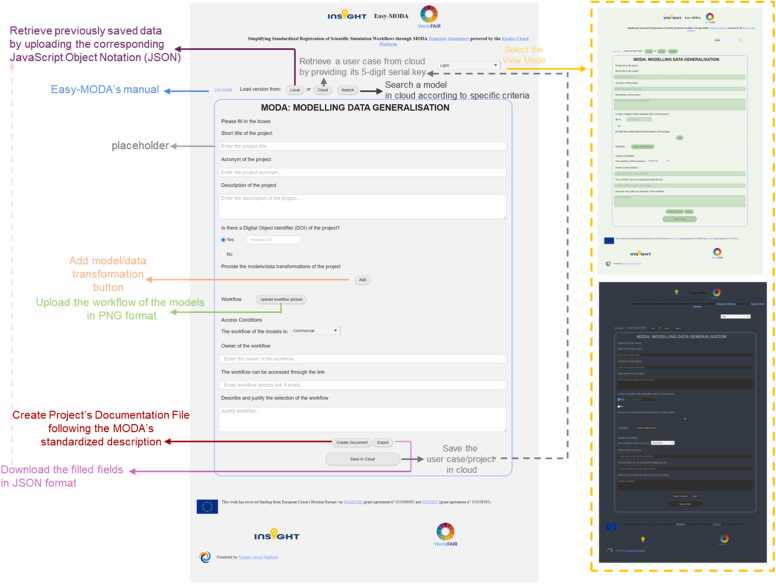
The Easy-MODA Platform's Graphical User Interface (GUI) is designed to be user friendly. It provides an initial form for entering general information about a project, its associated models, their access conditions, and workflow details. The platform visualises how the models are linked (if they are as not all models developed within a project have to be linked). Some models may function as stand-alone models.

Easy-MODA’s graphical user interface (GUI) is designed to streamline the collection of complex details of scientific studies that integrate different modelling practices and record this metadata in a structured format guided by the MODA framework. Easy-MODA equips researchers with features to comprehensively document their models from model or model-system overviews and modelling specifics to the digital object identifiers (DOIs) that ensure easy accessibility. The GUI of the Easy-MODA web tool, illustrated in Fig. 1, collects detailed information (metadata) about a specific model or set of models, that describe what has been modelled and how. Users are required to fill in all the necessary information to complete the OVERVIEW and the SIMULATION parts of MODA. The GUI provides a "User Guide" button for assistance and a "Load Version" feature to retrieve previously saved data in JavaScript Object Notation (JSON) format. Additionally, there is an option to switch visual background themes—light, green, and dark modes—for user comfort while working in Easy-MODA.

Easy-MODA applies a one-by-one matching with most of the fields of MODA templates but for some generic fields of MODA Easy-MODA requires more fields to be provided to support its underlying decision logic. This means that at least one field of Easy-MODA corresponds to each field of the MODA template. In this way, Easy-MODA guides the user exactly on the information to be inserted (see Easy-MODA description below for more details). For the same reason, the description of the fields of Easy-MODA differs slightly from the description of some of the fields of the MODA templates as Easy-MODA aims to make the instructions clearer to the user. Easy-MODA calls any set of collaborative actions used to address a specific use case a project, whether funded or not. The GUI initially prompts users to enter a "Short title of the project", a concise name that captures the essence of the use case. This is followed by an "Acronym of the project" field, a shorter, abbreviated form of the title if available, as reported in ref. [19]. The user then provides an overview of the project's aims and scope in the "Description of the project" field. Detailed instructions or prompts are provided within each text box to guide users on the type of information required. After this, the form asks for a "Digital Object Identifier (DOI)" for the project, a persistent identifier for electronic documents, if available. For EU projects this is the project number. DOIs can be created using the Zenodo platform (https://zenodo.org/records/51902) [21] or this field can be left blank.

**Fig. 2:**
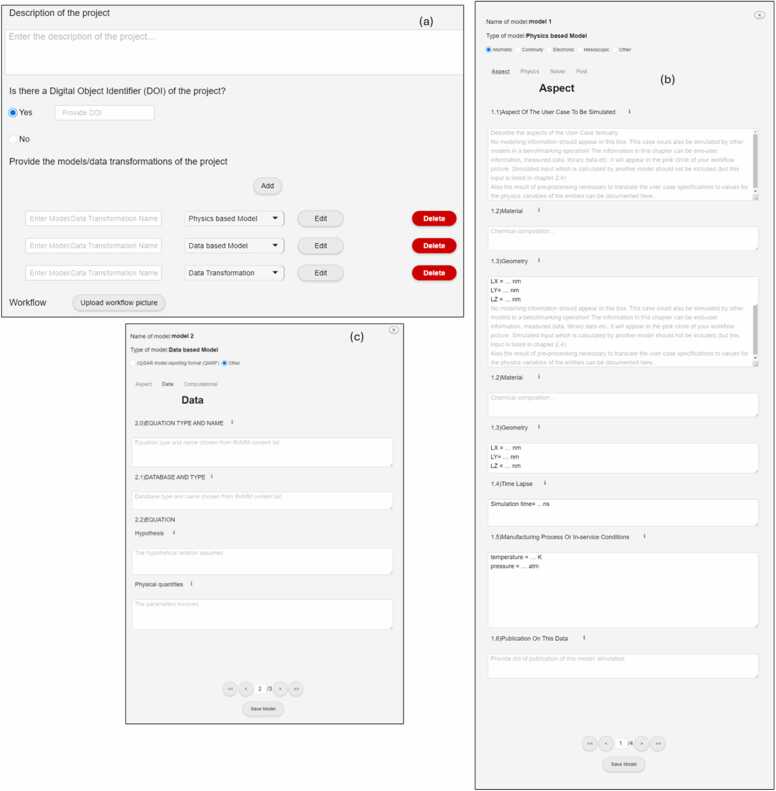
Overview of an example of the main GUI (a) and its input forms for 'Physics based' (b) and 'Data Based' (c) models on the Easy-MODA Platform, detailing the fields for model specifications, simulation parameters, and data integration.

**Fig. 3:**
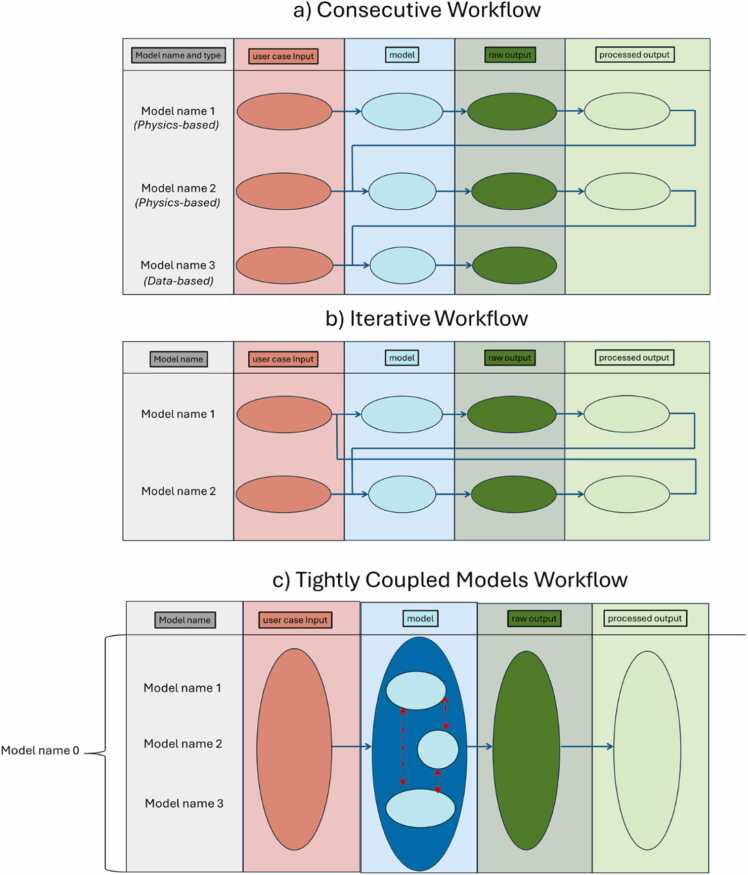
Consecutive (a), Iterative (b) and Tightly Coupled Models (c) Workflows.

Users are then asked to "Provide the models of the project" which can be done by clicking on the “Add Model” button (see Fig. 2-a) and adding its name. This action is repeated until all models associated with the project have been inserted. This action automatically creates new fields related to each model type, enhancing Easy-MODA’s capability compared to manual MODA template filling. This automation saves time and reduces the risk of errors, making EASY-MODA an efficient and reliable tool for managing model documentation (metadata). After inserting the model's name, the user defines the model type from a dropdown menu, choosing between "Data-based Model" and “Physics-based Model,” while having options for editing or deleting these models. The selection of a model type is crucial as it influences the available options that appear after clicking on the “Edit” button. Because the elaboration of model details is the most time-consuming stage of MODA, we recommend that users fill in the other fields of the GUI (as shown in Fig. 1), including the workflow image that describes how the models are linked, before editing each model as shown in Fig. 2. The user can use the workflow templates provided by EMMC to make its workflow image. These templates can be downloaded by clicking on the hyperlink “Template” at the top of Easy-MODA GUI (see Easy-MODA’s manual for more details) and provide consecutive, linked, iterative and tightly coupled model workflows (see Fig. 3).

**Fig. 4:**
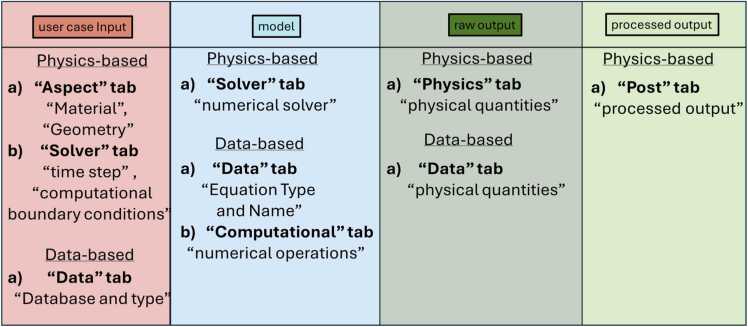
Easy-MODA fields that map to the workflow shapes according to the type of the model (i.e., physics-based or data-based). These fields consist of user case input, model, raw output and processed output (red, blue, dark green and light green boxes, respectively).

The workflow is divided into four zones (see Fig. 4). These zones are the use case input (red colour), the model (light blue colour), the raw output (dark green colour) and the postprocess output (light green colour). Every model has an object for each zone that needs to be described. The object in the red zone is filled with the information written in the Easy-MODA fields a) “Material” and “Geometry” of the “Aspect” tab that are to be simulated with the physics-based and/or data-based models, and b) “time step” and “computational boundary conditions” in the “Solver” tab (see below for more details) for physics based and “Database and Type” in the “Data” tab for data-based models. The object in the light blue zone records information written a) in the “numerical solver” field of the “Solver” tab for physics-based simulations and in the “Equation type and Name” field of the “Data” tab and b) the “numerical operations” field of the “Computational” tab for data-based models. The object in the dark green zone records information written in the field “physical quantities” of the “Physics” tab for the physics-based models, and in the “Data” tab for data-based models. The object in the light green zone records information written in the field of “processed output” of the “Post” tab for the physics-based models (see below for more details) while there is no such field for the data-based models. We plan to automate this procedure in the next version of Easy-MODA.

The "Access Conditions" section requires users to specify whether the workflow is based on commercial, free, or open-source software or models. If at least one workflow is based on a commercial software or model, the entire workflow is considered commercial. Free and open-source software and models are distinguished by whether the underlying code is accessible (open source) or just the model (free) [22].

In the "Owner of the workflow" field, users must provide information about the person or entity responsible for the workflow, including their email address (if possible) or their Open Researcher and Contributor ID (ORCIDs) thus documenting the model provenance. An additional input for "The workflow can be accessed through the link" field facilitates direct online access to the workflow, for example, through a web interface (see workflows in https://emmc.eu/moda/ for more) or through a DOI using the Zenodo [21] platform (https://zenodo.org/records/51902). Lastly, the section "Describe and justify the selection of the workflow" requires users to articulate the rationale behind choosing specific workflows and models, to provide context and aid others with interpreting the model outputs and facilitate re-use of the models. Upon completion of this first layer input, users can select "Create Document" or "Export," which allows the documentation file (DOC format) to be saved or facilitates external use of the data entered by saving it in JSON format, respectively. Saving and export can be done regardless of whether other fields are filled, allowing users to save their current status and upload them in the future through the “Load Version” button to continue filling in the MODA template. Furthermore, users can upload saved JSON files of older projects, or models / model combinations that resemble new ones, to modify them and create a MODA document from them. This makes the process easier for frequent users to document subsequent projects and models.

**Fig. 5:**
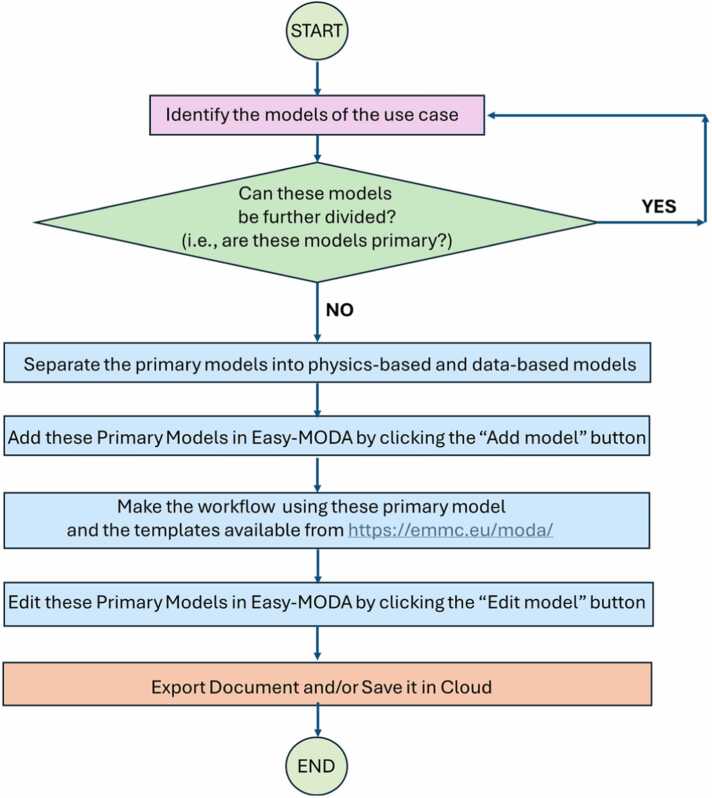
A flow chart illustrating the steps required prior to and during the use of Easy-MODA.

The MODA guidelines (see https://emmc.eu/moda/) divide models into two categories: a) physics-based models, where any physics equations are solved, and b) data-based models. However, in many user cases the description of models is not enough to describe precisely the whole procedure and a description of a pre- and post-process data transformation is often needed (e.g., construction of the initial configuration of a system (e.g., atomistic, coarse-grain, or macroscopic object) is considered a data transformations [23]). Because of this, Easy-MODA allows the user not only to insert the models of the whole procedure but also to insert any data transformation (e.g., constructing a nanoparticle (NP) from its CIF files by applying geometrical rules [20] is a type of data transformation) by providing its detailed description through a template that resembles to the template used for the data-based models by MODA. Easy-MODA aims to help the user divide their workflow into primary (i.e., they cannot be further split into smaller ones) data-based and physics-based models, even though these models are inextricably linked and interdependent (see Fig. 3). A primary model can be either a physics-based or a data-based model only. The interdependency between linked models is illustrated in Fig. 3(c), and the preparation steps before applying MODA are illustrated in the flowchart presented in Fig. 5. For instance, ASCOT [23], a web tool for the digital reconstruction of NPs, can be split into two primary models: a data-based one, which applies geometrical operations to a CIF file to create a neutral spherical NP; and a physics-based one, which applies energy minimization to the NP to generate its structure. Calculation of atomistic descriptors by ASCOT is considered the post-processing part of the physics-based model. In cases where complex transformations of the physical properties derived from the energy minimization are applied, the calculation of atomistic descriptors can be treated as separate data-based models to allow the user to describe these transformations comprehensively.

Each model is added individually in Easy-MODA and then it is coupled with the models already present (see models coupling in Fig. 3). To input information for each model declared by a project, the user clicks on the *“Edit”* button of Fig. 2(a) and the pop-up window of Fig. 2(b) appears when the model is physics-based, or the pop-up window of Fig. 2(c) appears in case of a data-based model. Fig. 2(a) illustrates the GUI of a physics-based simulation model, with an example model named "Atomistic Descriptors Calculation" which describes the model used by Varsou et al. [20] for the calculation of atomistic descriptors. At the top of Fig. 2(a), the interface confirms the model's classification as 'Physics based'. Users then refine the model's entity by choosing from 'Atomistic’, ‘Continuity', 'Electronic', 'Mesoscopic' or 'Other'. The selection of the model entity creates pre-filled fields in the Easy-MODA template by mentioning all the available options. The user can then erase the options that are not included in their model and fill in any option that may be missed. These options are further organised into groups for the different methodologies corresponding to the model entity (i.e., *'Atomistic', 'Continuity', 'Electronic', 'Mesoscopic' or ‘Other’*). Each methodology appears in brackets and its options are under them to guide the user (e.g., Molecular Dynamics, Monte Carlo, and Energy Minimization are options for the Atomistic Model category). Easy-MODA has also the option ‘Other’ for any physics-based model which cannot be considered as atomistic, continuous, electronic or mesoscopic. The Physiologically Based Kinetic (PBK) model can be such a model because it contains a set of ordinary differential equations of first order where a solver (information) is needed [15]. This approach agrees with the MODA documentation created in the past for the PBK model for the calculation of the accumulated nanomaterial mass in the human respiratory system [15]. Easy-MODA has prefilled fields for the PBK models based on the OECD PBK Model Reporting Template [24] to help users to fill MODA fields easier(see Table 3 of ref. [24]).

For physics-based model characteristic selections, the interface is organised into "Aspect", "Physics", "Solver", and "Post" tabs (see Fig. 2(a). In this instance, the "Aspect" tab is active, indicating that the user is currently entering details pertinent to this stage of the model's setup. The other tabs, when selected, allow the user to add the governing physical laws for the model ("Physics"), computational techniques and algorithms to solve the model equations ("Solver"), and the analysis of simulation outputs ("Post"). The interface's design facilitates smooth navigation through these tabs, with controls at the bottom of the form. As users navigate between tabs, they encounter specific fields and guidance pertinent to each section. This segmentation ensures that the process of defining the model is methodical and comprehensive, with each tab focusing on a different aspect of the simulation's framework. In addition, the user can get further instructions through examples by moving the mouse cursor onto the *“i”* (for information). The first section, *"Aspect of The User Case To Be Simulated"*, instructs users to describe the scenario or case that the model aims to simulate. Here, the Easy-MODA platform emphasises that the description should be textual and should avoid including any calculated data or end-user information that might be processed by another model.

In the "Material" subsection, users are instructed to provide the chemical composition of their simulated system. For the case study of Varsou *et al.,* Ag, TiO_2_, and CuO NPs are the selected materials, as described by specific CIF files (i.e., space group, unit cell dimensions, fractional coordinates) characterised by a unique identification (ID) number in the Crystallography Open Database (COD) or the DOI of the related publication. The relevant COD ID numbers and the procedure for initial construction of the configuration files of the materials should be included here. For example, in the case study of Varsou et al., the ASCOT web tool was used to get geometrically constructed NPs as the initial configuration. Similarly, any parameters that were used to build the initial configuration of the simulated system with its selected options should be mentioned so that the simulated system can be reproduced in the future. For instance, for a polymer builder, such parameters could include polymer entanglement average length, dispersity index, characteristic ratio etc. Alternatively, the user can provide the file with the atomic coordinates at the end of the MODA metadata file as an appendix or a hyperlink to the specific file. This building procedure is extremely important for amorphous materials where their structure cannot be obtained from X-ray diffraction (XRD) data (single crystal or Rietveld Refinement method). For crystalline materials, if the CIF file has not been retrieved from a database but was created from XRD data analysis during an experimental project, a data transformation/analysis is needed (see above) to make the configuration files needed as input from the physics-based models. The output from this transformation/analysis is the input to the physics-based model. For example, if Rietveld Refinement was applied for the XRD data analysis, then the software used shall be mentioned (e.g., Fullprof [25]) along with the metrics used to investigate the fit of the experimental data to the Rietveld mathematical model (e.g., Durbin Watson metric, expected R-value, profile R-value, weighted profile R-value, Bragg R-value, goodness of fit, etc.), the fitting method (e.g., least squared error) and the model parameters (e.g., Cagliotti function, pseudo-Voigt mixing parameter, asymmetry parameters, zero shift, etc.).

To interpret the results of the workflow and investigate its reliability, each workflow model should be described as comprehensively as possible. In case an unpublished in-house code is used for a physics-based or a data-based model, the user must describe the procedure of the code in the “*Computational*” subsection in the field “*Numerical Operations*”. For electronic, atomistic, and coarse-grained models, the material’s phase should also be named to avoid any confusion, as different phases have the same chemical formula (e.g., TiO_2_ which can be found in rutile, anatase, and other phases in nature)*.* The more information added into the fields, the richer and more complete the metadata for the model, thus, the more accurate and reproducible the model will be. This requirement for rich metadata to support model interoperability and reusability provides the impetus for filling the following fields. The *“Geometry”* field requests specific data about the geometry of the system to be modelled (e.g. size, form, drawing, picture of the system) and it should not be confused with the computational domain which is provided in the “Solver” tab of the physics-based model’s popup window through the “Computational Boundary Conditions” field. For the case study of Varsou et al. [20], the diameters of the NP investigated need to be declared in this field.

In the “Time Lapse” field, users provide details about simulation time (i.e., the duration in real world of the situation to be simulated which differs from the Central Processing Unit (CPU) time which is the amount of time a program is running to do the simulation) and should not be confused with the computational wall-clock time or time step that are provided in the “Solver” tab and its “Time Step” field. Discrete steps and simulation time are not always proportional, (e.g., for kinetic Monte Carlo simulations the time step value varies from step to step). For energy minimization (such as the study of Varsou et al., [20]) the “Time Lapse” field can remain empty.

A more complex subsection is "Manufacturing Process or In-service Conditions", which encompasses several checkboxes and fields and is not about the simulation itself, but the user case (i.e. the conditions in real world that are to be simulated such as pressure, temperature, concentration profiles, etc.). For the physics-based model used in the study of Varsou et al., [20] no the concentration, the shape, the size and the type of the nanoparticles as well as the pH and any information about the dispersion medium is the information that should be included in "Manufacturing Process or In-service Conditions" field.

Lastly, "Publication Of These Data" provides a space for users to link the simulation to its scholarly publication by entering the DOI associated with the model (e.g., a publication that explains the procedure for the extraction of CIF) or at least to provide a link for their CAD file for continuum simulations. The "Save Model" button allows the user to save the filled fields temporarily before moving to another tab using the navigation controls at the bottom. However, the user needs to move to the main MODA GUI and click on "Export” to save the project’s data, including the model’s data.

Moving to the “Physics” tab, the entries of “Model Category” and “Model entity” are automatically filled when the physics-based simulation category (i.e., Atomistic, Continuity, Electronic and Mesoscopic) was selected. The field of “Model Equations” is filled according to this preselection. The user can erase any Model Equation that has not been used in its model or manually add any other in case not present. The physical quantities used in the model’s equations are declared in the field of “Physical quantities”. Any equation/function that describes the coefficients such as viscosity, thermal conductivity and heat capacity for continuum models, force-fields used for atomistic models, and basis sets and exchange correlation functionals for electronic models used in the model’s equation should be declared in the field of “Materials relation”.

Describing the simulation system, its initial conditions, and its equations, is not sufficient to ensure the reproducibility of the results as two additional stages, Solver and post-process, are required. The “Solver” stage describes the algorithms used to solve the equations such as finite elements for continuum models [26], velocity Verlet for molecular dynamics [27], conjugate gradient for energy minimization [28], or self-consistent field for electronic models [28]. Additionally, some solver parameters that are critical for accuracy and interpretability of the results, (e.g., force-field short-range and long-range cutoff [27], [28], neighbour list frequency and neighbour list cutoff for atomistic models [27], [29], grid size, grid type and grid thickness for continuum models [30], etc.) are provided in the “Additional Solver Parameters” field. Different solvers can lead to markedly different structures or results because of their high sensitivity to the input parameters (e.g., structure optimizations may be trapped in local minima, the distance cutoff for long-range interactions can affect the results for atomistic simulations, while the thickness of the mesh and the Courant number [31], [32] is very important for continuity models to assure the stability of the solver’ solution). The numerical stability of the solver is also important and information relating to propagation of numerical errors is essential for all the types of simulations (electronic, atomistic, coarse grain, continuity). For quantifying numerical stability, the user can use the Von Neumann stability analysis [32]. In addition to these fields, the software used for this model is also crucial due to latent options in each software package that are not visible to users and are therefore not declared in previous fields. The reference to software employed provides extra security in case of missing information in the declared fields.

The Post-Process Stage is also essential as it describes how the user treats the primary outcomes of the model to calculate properties depending on them. The Methodologies are based on statistical analysis of the primary output (e.g., minimum, maximum, and average value of a physical property profile derived by a continuum model, mean square displacement of molecules and autocorrelation functions of physical properties for atomistic models, electrostatic potential fit and electron density population analysis for electronic models, etc.). The field “Margin of Error” describes how this is calculated, for example, standard deviation, box plots, confidence interval upper and lower limit.

Similarly, Fig. 2(b) illustrates the user interface for documenting a data-based simulation model where the main functionalities remain the same. In contrast to physics-based models with tabs named "Aspect", "Physics", "Solver", and "Post", data-based models are organised by “Aspect”, “Data” and “Computational” tabs. The “Aspect” tab is similar to the corresponding tab of physics-based models. In the “Manufacturing process or in-service conditions” field of the tab, the data produced by physical experiments can be described extensively and integrated with other workflows made for experimental procedures. For the case study of Varsou et al. [20] the NP synthesis procedure, the process for the measurement of the NP physico-chemical properties, and the postprocessing of these properties to create the data in the dataset (e.g., mean value, standard deviation, etc.) is added to this field. In the case of computer experiments (e.g., the atomistic descriptors for the case study of Varsou et al. [20]), a reference to another model developed within the project is needed. If data has been retrieved from a database that satisfies the FAIR principles, a reference to the dataset with its ID and access date is sufficient. The Equation type can be selected from a variety of widely-used machine learning algorithms: e.g., convolutional neural network [33], long-short term memory network [34], recurrent neural network [35], [36], [37], decision tree [37], k-means [38], k nearest neighbours (kNN) [39], logistic regression and other non-linear least squares mathematical functions (the parameters of which are found by minimising a cost function).

The QMRF template proposed by the OECD (https://www.oecd.org/chemicalsafety/risk-assessment/qsar-assessment-framework-annex-1-qsar-model-reporting-format.docx) has been also included in Easy-MODA to add documentation options for each method that ensure the reproducibility of the results. According to the OECD [40], a QSAR model should satisfy the following properties: a) a defined endpoint, b) a defined domain of applicability, c) an unambiguous algorithm, d) appropriate measures of goodness-of–fit, robustness and predictivity and e) a mechanistic interpretation, if possible. The first property (i.e., a defined endpoint) is added to the “Aspect of the User Case to be calculated” field in which guidelines are added as a placeholder that are removed when relevant text is added. If there is no information about fields such as “Geometry” and “Time Lapse”, these fields can remain empty. Applicability domain information is added via the “Manufacturing process or in service conditions” section. This guides the user to add information about the range of the descriptors values for which this model is reliable, how the training set is distributed in the space and how it was selected, and the method used to evaluate the model’s applicability domain. Besides the training set information, the same information is required when a specific mathematical function is selected to model the data. The third essential QSAR property, an unambiguous algorithm, is described in the field “Equation type and name”. Statistical and machine learning algorithms such as k-nearest neighbours [39], decision trees [37], support vector machines [40], partial least square [41], principal component analysis [42], random forests [43], k-means clustering [38], XGBoost [44], naïve Bayes [45], convolutional neural networks [33], recurrent neural networks [46], long-short term memory networks [34]) or analytical mathematical functions are available options. The user can also define new options. The fourth essential QSAR property, appropriate measures of goodness-of–fit, robustness and predictivity, is documented in the field “database and type”. Here, the selected training set should be indicated by a link (DOI for the dataset) to enable others to reproduce the metrics of the model. If the training set cannot be shared because it is proprietary, this should be mentioned. However, this clearly hampers the reusability of the model. If design of experiments (DoE) has been used to create a training set, the DoE algorithm used should be mentioned (e.g., Latin hypercube sampling [47], orthogonal design [47], optimal experimental design [48], etc.). The metrics for the goodness of fit should be provided in the field “margin of error”. In the field “Software tools”, a list of libraries and software that are widely used for data-based models are listed, such as KNIME [49], tensorflow [50], etc. and users can add other metrics if needed. In the field “numerical operations”, any correlation between different integrated data-based methodologies can be listed.

### 2.1 Easy-MODA workflow

**Fig. 6:**
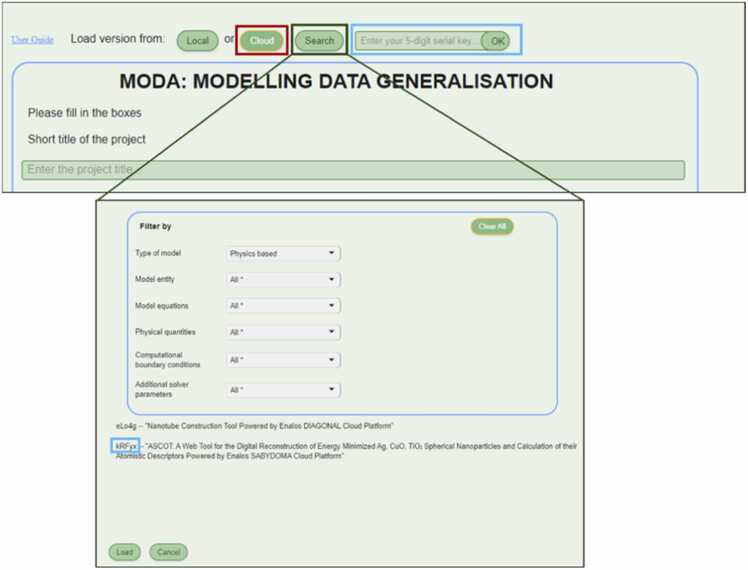
Easy-MODA acts as a registry of MODA use cases. The available MODA use cases are accessible through the “Cloud button” (red box). Every cloud use case can be selected by inserting the unique 5-digit serial key of the use case. A search of the use cases according to user-defined criteria is also available through the “Search” button and a pop-up window appears at the bottom of the screen.

Easy-MODA can also save each use case/project to a cloud for later use. A five-digit serial key is assigned for each instance in the cloud which can be used to retrieve its MODA documentation (see Fig. 6). A “Search” button has also been included in Easy-MODA’s GUI to enable search of the use cases that satisfy criteria defined by the user. In this way, Easy-MODA not only simplifies the harmonisation of documentation within the MODA guidelines, but also serves as a registry of the use cases, making them Findable and increasing their compliance with the FAIR principles. Cloud registration accelerates MODA documentation of new use cases/projects based on existing use cases because only a modification of an existing case is required.

Easy-MODA has been included into the Enalos Cloud Platform to facilitate documentation of the platform’s predictive tools and molecular builders, and re-use of the metadata where these models are integrated to workflows. Offering MODA as a webtool on the Enalos Cloud Platform is a strong indication that the platform aims to develop, implement, and facilitate workflows with greater transparency, reproducibility, and FAIRness, and drive regulatory acceptance of nanoinformatics and materials modelling approaches. Next, we present a demonstration of the Easy-MODA workflow as applied to the ASCOT (https://www.enaloscloud.novamechanics.com/sabydoma/ascot/) and SafeNanoScope (https://www.enaloscloud.novamechanics.com/sabydoma/safenanoscope/) tools of the Enalos Cloud Platform.

### 2.2 Test case demonstrating the Easy-MODA workflow for integrated models

The application of the Easy-MODA tool to a modelling workflow for an integrated approach to toxicity testing and assessment (IATA) reported by Varsou et al. [20] is described. This modelling workflow showcases the advantages of linking ASCOT [23] with autoML and synthetic data creation using MODA's systematic and unified documentation processes, as formalised in the Easy-MODA tool. This model combination is designed to enhance the reproducibility and ease of access to nanotoxicity datasets. It achieves this by enriching experimental datasets with calculated NP descriptors and fully *in silico* data using digitally constructed NPs. The ultimate goal is to reduce reliance on animal testing in the future.

### 2.3 Enhancing nanotoxicity evaluations through systematic documentation

**Fig. 7:**
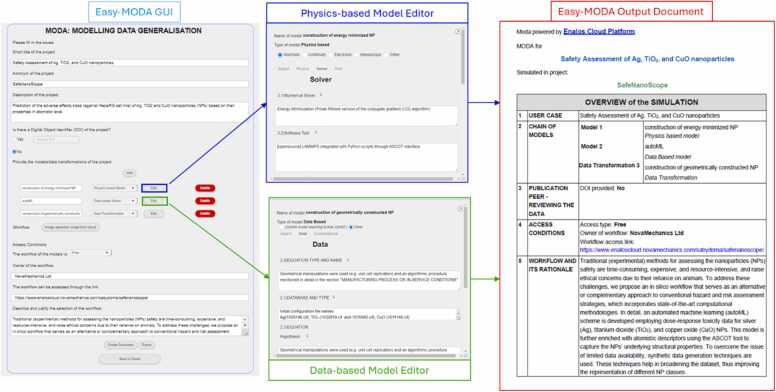
The Easy-MODA Interface Workflow from Input to Output Documentation. The image captures the step-by-step process of using the Easy-MODA system, starting from data entry in the GUI, through the editing modules for physics-based and data-based models, to the final output of a harmonised MODA document. This output encapsulates the project overview and simulation details, showcasing the system's efficacy in automating and documenting materials modelling workflows represented here by a NP toxicity simulation.

Fig. 7 demonstrates the systematic approach taken in the Easy-MODA platform to model data generalisation, which is central to our test case scenario. The left panel of Fig. 7 shows the Easy-MODA GUI, where the user initiates the modelling process. Here, detailed project information is entered, including project title, acronym, description, and details of the models to be used—whether physics-based, relying on fundamental principles, or data-based, utilising empirical datasets. This demonstration case comprises both a physics-based model and a data-based model. As a final outcome from the combination of these models, we predict the adverse effects class (non-toxic or toxic) of Ag, TiO_2_, and CuO NPs to HepaRG cells based on their properties at the atomic level [20].

**Fig. 8:**
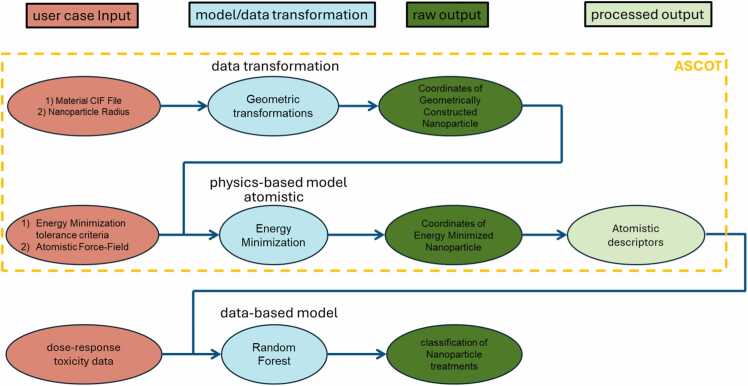
Workflow diagram illustrating two simulation pathways. The upper pathway represents an atomistic model using a Material CIF File input to execute a conjugate gradient energy minimization method (a data-driven approach), yielding atomic coordinates which are processed into atomistic descriptors via a physics-based atomistic model. The lower pathway shows a data-based model that inputs dose-response toxicity data and the atomistic nanoparticle (NP) descriptors into a Random Forest model to classify NP treatments as toxic or non-toxic.

The workflow diagram presented in Fig. 8 shows the simulation pathways embodied by the test case. The upper pathway follows a data-based model that uses a Material CIF File as input to apply geometric transformations to find the coordinates of the atoms of a geometrically reconstructed NP. In the middle pathway, these coordinates are used as input to the next model where a conjugate gradient energy minimization method (a physics-based model) is executed, resulting in raw interatomic distances which are then processed into atomistic descriptors. This middle pathway exemplifies the application of physics-based simulations to derive fundamental insights into the structural properties of NPs and their implications on toxicity. Conversely, the lower pathway illustrates a data-based model that leverages dose-response toxicity data (and the atomistic descriptors from the physics-based model) as inputs into a Random Forest model, enabling the classification of NP treatments as toxic or non-toxic. This approach highlights the incorporation of machine learning techniques to predict NP toxicity, leveraging empirical data to inform safety assessments. Such a data-driven approach is instrumental in identifying patterns and relationships that may not be apparent through traditional analysis methods.

The documentation of the workflow of Fig. 8 created by Easy-MODA is available in the Supporting Information.

### 2.4 Limitations of Easy-MODA currently, and future developments to address these

A limitation in the current version of EasyMODA is the absence of an automated way to create the workflow image. This limitation and how the user can work around it are described in the user manual of Easy-MODA. Future versions of Easy-MODA will address this limitation as a priority. Examples of computational fluid dynamics (CFD) and quantum chemistry models, as well as multiscale modelling workflows that have the information of CFD and quantum chemistry are currently being added to Easy-MODA and will be available via the cloud platform in the near future starting with the projects already documented following the MODA guidelines and available in Ref. 12. Another limitation currently is the absence of guidelines on how to complete the MODA for omics based models (proteomics, transcriptomics, Adverse-Outcome Pathway etc.), but this is beyond the scope of this paper and requires consensus from scientists and regulators on what information is needed for omics-based models which can then be formalised as a set of required fields and guidance on how to fill these fields via Easy-MODA. Because Easy-MODA will evolve in time, these limitations are expected to disappear in the near future.

## 3 Regulatory outlook for Easy-MODA

We have seen earlier in this paper how QSAR and PBK models can be written in the format of MODA templates following the OECD guidelines [10], [24] using Easy-MODA. In the future, the MODA concept may be applied/extended to many other modelling integration challenges such as integrating data from experimental non-animal-methods as well as omics-data interpretation models.

The available OECD model reporting templates for QSAR model [10], PBK models [24], and in vitro models (evolved with Krebs et al. [51]) vary in their grouping and naming of information requirements, though all of them ask for information about the same conceptual criteria of validity. A regulatory assessment of the validity of integrated models may become easier via the definition of conceptual validity criteria which are relevant for any type of model, and which are then attributed to the various specific lines in the model reporting templates. This may support a conceptual understanding of the various information types within the MODA and their utility for the assessment of integrated model validity. Technically this attribution of conceptual validity criteria could be implemented within the OECD formats and/or within Easy-MODA. We may base the definition of five conceptual validity criteria on the modular concept of validation (Hartung et al. [52]), included within the OECD Guidance document on model validation [53] and refine the terminology for an intuitive understanding of the underlying validity concepts for experts coming from various different fields: 1) FAIR model identity; 2) technical and/or regulatory purpose; 3) relevance and/or, correctness, precision, scientific trustworthiness, robustness; 4) reliability and/or variability upon replication; 5) applicability domain (AD) upstream and/or downstream. In the Supporting Information of this publication, we provide some explanations for these five conceptual validity criteria. In addition, we provide figures suggesting how the information requirements within the OECD model reporting templates could be attributed with these five criteria and how they could be related to the MODA format and implemented in Easy-MODA. We will discuss and further explore this approach and possibly extend it to omics-data-interpretation models beyond the currently available omics-data-reporting formats [54]. This may prove useful for the INSIGHT case studies aiming for a potential regulatory assessment and use of integrated models at OECD level. A detailed explanation about how the underlying automation in Easy-MODA works is illustrated in the flowchart of Fig. S2 in the Supporting Information.

## 4 Conclusion

Easy-MODA, a web tool hosted in the Enalos Cloud Platform, provides a structured and systematic approach to guide users through the process for documentation of complex simulation workflows. By filling each of the Easy-MODA fields stepwise, it ensures that every relevant detail is captured in a harmonised and standardised manner, enhancing clarity and consistency across the scientific community and improving model interoperability and reusability (consistent with the FAIR principles). This uniform approach to documentation of simulations and computational workflows is embedded in a Software as a Service (SaaS) platform. It paves the way for seamless collaboration, review, and validation processes for nanoinformatics and materials models that are integral to scientific advance. The enforced structure and the available options for documentation of physics-based and data-based models within Easy-MODA have been described and exemplified in a case study. The selection of the model type and entity leads to pre-population of some fields and a reduction of the number of available options, thereby streamlining and simplifying the process of completing the Easy-MODA fields and reducing the risk of error.

We have shown that the separation of a complex modelling workflow into its primary models, and definition of materials relations are the critical parts of creating a successful MODA documentation. This is because of the presence of many “hidden” data-based models (e.g., any model used for the data transformation procedure to create the initial configuration of the simulated system). Similarly, the post-processing of a physics-based model can include hidden data-based models in cases where complicated data transformation occurs. Bringing these formerly hidden steps to light enhances (regulatory and industry) confidence in the models, reduces the “black-box” concerns around *in silico* approaches, and enhances the re-usability of the models into IATA workflows. Registration of use cases/projects (MODA-based metadata regarding the models) is achieved in a cloud from which the use cases can be retrieved and searched according to user-defined criteria. This capability is essential for compliance of the Easy-MODA documentation with the FAIR principles, and ensures that the metadata remains available even if the model is no longer available.

To illustrate how Easy-MODA simplifies MODA documentation and provides guidance for completion of MODA templates, we applied it to the workflow described by Varsou et al. [20], which models the safety of Ag, CuO, and TiO_2_ NPs. Three primary models (two data-based and one physics-based model) were incorporated, documented, and visualised using the Easy-MODA web-application. The resulting downloadable and shareable model documentation, prepared according to the MODA principles, is available in the Supporting Information (Easy-MODA manual). Several extensions to Easy-MODA are planned and will be reported in due course.

The flexibility of Easy-MODA offers users the ability to customise fields as needed, to modify or expand the pre-filled fields to document unconventional workflows. While our primary focus here was on nano-informatics, Easy-MODA’s design inherently supports a variety of computational fields including computational fluid dynamics, quantum chemistry, computational biochemistry, thanks to its ability to handle both physics-based and data-based models.

## Funding

This work was funded by the European Union’s Horizon Europe Research and Innovation Programme via the WorldFAIR project (grant agreement nº 101058393) and the INSIGHT project (grant agreement nº 101137742) including the 10.13039/100014013UKRI Guarantee Funding for UoB’s contributions to WorldFAIR (1831977) and INSIGHT (10097888). Development of the models on which EASY-MODA is demonstrated was funded via the European Union’s H2020 Research and Innovation Programme through the SABYDOMA project (grant agreement nº 862296) and the 10.13039/100010665H2020 Marie Skłodowska-Curie Actions project CompSafeNano (grant agreement nº 101008099). TEE acknowledges Horizon Europe Research and Innovation Programme project PINK (grant agreement nº 101137809) including the UKRI Guarantee Funding for the UoB contribution (10097944). GG acknowledges support from the OpenModel project which has received funding from the European Union's Horizon 2020 research and innovation programme under grant agreement No 953167. The work of MP at the Medical University is co-financed via 10.13039/100006672PARC(101057014) and the Austrian Federal Ministry for Climate Action, Environment, Energy, Mobility, Innovation and Technology, Department V/5—Chemicals Policy and Biocides.

## CRediT authorship contribution statement

**Panagiotis D. Kolokathis:** Methodology, Writing – original draft. **Nikolaos K. Sidiropoulos:** Software. **Dimitrios Zouraris:** Methodology, Writing – original draft. **Dimitra-Danai Varsou:** Writing – original draft. **Dimitris G. Mintis:** Writing – original draft. **Andreas Tsoumanis:** Software. **Francesco Dondero:** Writing – review. **Thomas E. Exner:** Writing – original draft, Writing – review, Funding acquisition. **Haralambos Sarimveis:** Writing – original draft. **Evgenia Chaideftou:** Writing – original draft, Writing – review. **Martin Paparella:** Writing – original draft, Writing – review, Funding acquisition. **Fotini Nikiforou:** Writing – review. **Achilleas Karakoltzidis:** Writing – review. **Spyros Karakitsios:** Writing – review. **Dimosthenis Sarigiannis:** Writing – review. **Jesper Friis:** Writing – original draft, Writing – review. **Gerhard Goldbeck:** Writing – original draft, Writing – review. **David A. Winkler:** Writing – review. **Willie Peijnenburg:** Writing – review. **Angela Serra:** Writing – review. **Dario Greco:** Writing – review, Funding acquisition. **Georgia Melagraki:** Supervision. **Iseult Lynch:** Conceptualization, Supervision, Writing – original draft, Funding acquisition. **Antreas Afantitis:** Conceptualization, Methodology, Supervision, Writing – original draft, Funding acquisition.

## Declaration of Competing Interest

PDK, NS, DZ, DDV, DM, AT, AA are affiliated with NovaMechanics, a cheminformatics and materials informatics company.

## Footnotes

Appendix A Supplementary data associated with this article can be found in the online version at doi:10.1016/j.csbj.2024.10.018.
