# Supplementary material for: Easy-MODA: Simplifying standardised registration of scientific simulation workflows through MODA template guidelines powered by the Enalos Cloud Platform
Source: Comput Struct Biotechnol J. 2024 Oct 18;25:256–68. doi: 10.1016/j.csbj.2024.10.018 (PMC11566491; doi:10.1016/j.csbj.2024.10.018)
Supplement: Supplementary file 1 — Supplementary material [file mmc1.pdf]

Supporting Information for the paper:

Easy-MODA: Simplifying Standardized Registration of Scientific Simulation Workflows through MODA Template Guidelines powered by the Enalos Cloud Platform

Panagiotis D. Kolokathis[a,b], Nikolaos K. Sidiropoulos[a,b], Dimitrios Zouraris[b,c], Dimitra-Danai Varsou[a,b], Dimitris G. Mintis[b,c], Andreas Tsoumanis[a,b], Francesco Dondero[b,d], Thomas E. Exner[e], Haralambos Sarimveis[f], Evgenia Chaideftou[g], Martin Paparella[g], Fotini Nikiforou[h], Achilleas Karakoltzidis[h], Spyros Karakitsios[h], Dimosthenis Sarigiannis[h], Jesper Friis[i,j], Gerhard Goldbeck[j,k], David A. Winkler[l,m,n], Willie Peijnenburg[o,p], Angela Serra[q], Dario Greco[q], Georgia Melagraki[r], Iseult Lynch[b,s,*], Antreas Afantitis[a,b,c,*]

[a]NovaMechanics MIKE, Piraeus 18545, Greece
[b]Entelos Institute, Larnaca 6059, Cyprus
[c]NovaMechanics Ltd, Nicosia 1070, Cyprus
[d]Department of Science and Technological Innovation, Università del Piemonte Orientale, 15121 Alessandria, Italy
[e]SevenPastNine GmbH, Rebacker 68, 79650 Schopfheim, Germany
[f]School of Chemical Engineering, National Technical University of Athens, 15780 Zografou, Greece
[g]Department of Medical Biochemistry, Medical University of Innsbruck, Innsbruck,Austria
[h]Aristotle University of Thessaloniki, Department of Chemical Engineering, Environmental Engineering Laboratory, University Campus, Thessaloniki 54124, Greece
[i]Department of Materials and Nanotechnology, SINTEF Industry, Trondheim N-7465, Norway
[j]European Materials Modelling Council, Brussels 1050, Belgium
[k]Goldbeck Consulting Limited, Cambridge CB4 0WS, UK
[l]La Trobe Institute for Molecular Science, La Trobe University, Bundoora, Australia
[m]Monash Institute of Pharmaceutical Sciences, Monash University, Parkville, Australia
[n]School of Pharmacy, University of Nottingham, Nottingham, United Kingdom
[o]Institute of Environmental Sciences (CML), Leiden University, Leiden 2300 RA, The Netherlands
[p]Centre for Safety of Substances and Products, National Institute of Public Health and the Environment (RIVM), Bilthoven 3720
[q]FHAIVE, Faculty of Medicine and Health Technology, Tampere University
[r]Division of Physical Sciences and Applications, Hellenic Military Academy, Vari 16672, Greece
[s]School of Geography, Earth and Environmental Sciences, University of Birmingham, Birmingham B15 2TT, United Kingdom BA

Corresponding authors: afantitis@novamechanics.com (Antreas Afantitis) and i.lynch@bham.ac.uk (Iseult Lynch)

Suggestion of conceptual validity criteria applicable for all types of (chemoinformatics and nanoinformatics) models:

1) **FAIR model identity**

   The model must be findable, accessible, interoperable and its development must be reproducible as a basis for re-usability. See for example Lynch and Afantitis, 2024 for details on how to make nanoinformatics models FAIR.

2) **Purpose of the model in technical and/or regulatory terms**

   A quantitative structure-activity relationship (QSAR) model could be used for technically estimating water solubility, predicting the lethal concentration for 50% of organisms (an LC50 value) such as fish or as the basis for a regulatory classification and labelling via the Globally Harmonised System (GHS), for instance.

3) **Relevance and/or correctness, precision, scientific trustworthiness, robustness**

   Depending on the model and the available data and information, the relevance of a model for a specific purpose or question can be determined by data-correlation between different models, including established reference models. Such data-correlation may be expressed quantitatively for correct/identical categorical results (e.g., toxic versus non-toxic, binding versus non-binding, gene expression change versus no change) or precision for continuous data. In addition, relevance may be expressed in terms of a mechanistic understanding of the aspect of the system / interaction modelled and its relation to the aspect that shall be regulated. Scientific trustworthiness is a conceptually similar term and may be more intuitive for omics data modellers who typically rely on different quality assessed databases for gene to pathway annotation, for instance. Robustness means potential variability stemming from slight modifications of the experimental model set-up or procedure (within the frame of the Standard Operating Procedure) or computational model development.

4) **Reliability and/or variability upon replication**

   Within the context of validation of *in vitro* models the term reliability is related to the variability of results upon replication of the experimental procedure within the same laboratory or between laboratories. For computational models this conceptual validity category is usually not relevant. The exception may be some complex models including Monte-Carlo simulations or other aspects that may introduce variable outcomes. Recently, the approach of consensus modelling has been implemented as a means to assess the reliability of predictions from several models each using a different modelling approach in order to reach a consensus prediction (e.g., Varsou et al., 2024), alongside the standard statistical approaches to confirm prediction reliability.

5) **Applicability domain (AD) upstream and/or downstream**

   ADs are used as a gate keeper, to define what data should not be used as input for the model or what model output should not be used further as the predictions are unreliable. For QSARs the upstream AD may be defined as a chemical structural space of the training data set used within the model development and model

validation and the downstream AD could be defined by rules for the acceptance of data based on similar model results for the structurally closest neighbours. For gene to pathway annotation and benchmark concentration (BMC) modelling using omics data, an upstream AD could be defined for example via a minimum number of replicates and doses and a downstream AD in terms of acceptable confidence intervals to BMC.

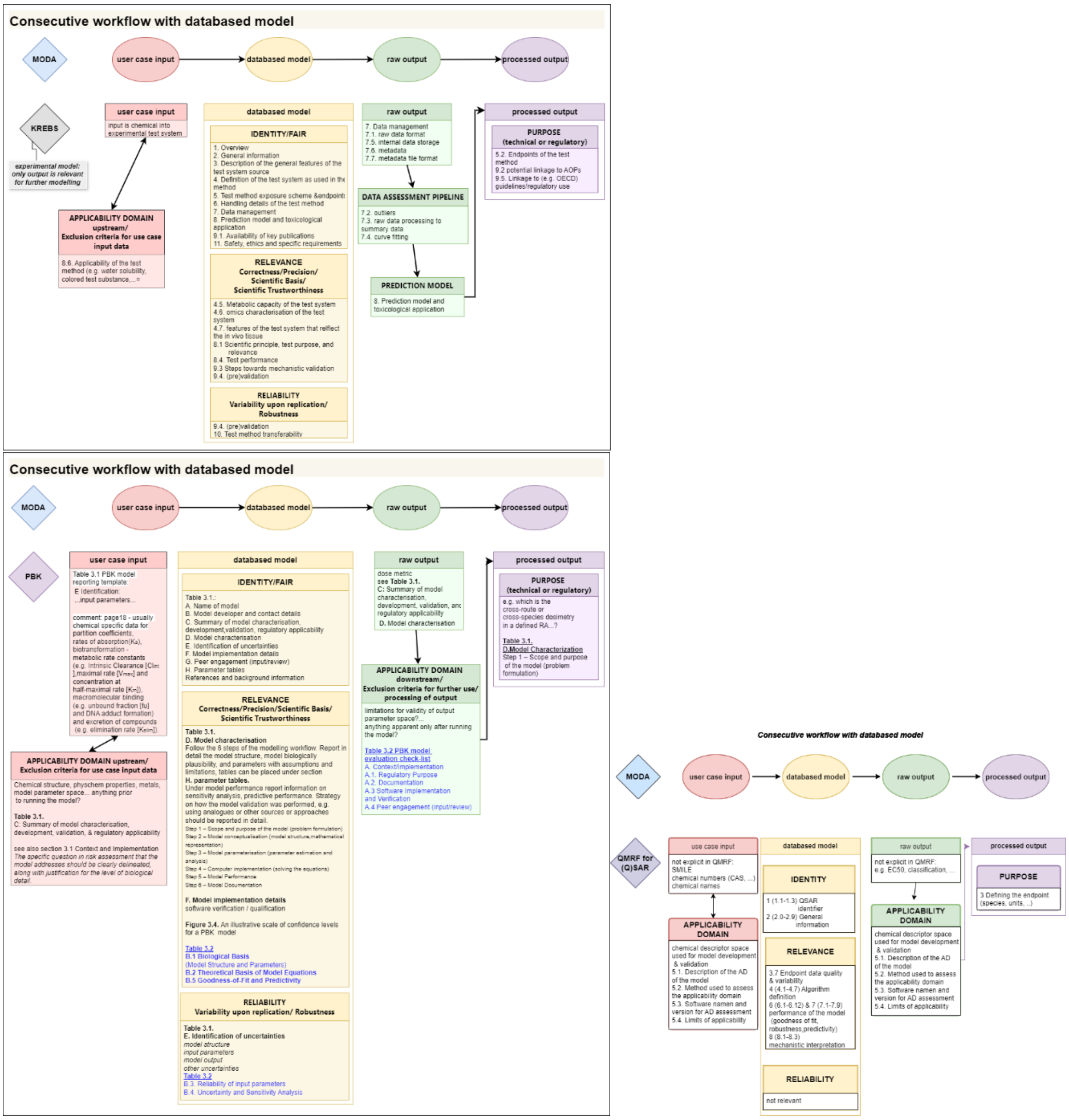

Figure S1: Suggestion regarding how the overarching conceptual validity criteria for models (as defined above) may be linked to the current OECD model reporting formats for QSARs, PBK models and experimental non-animal-methods. The headings and their numbering provided in the boxes for each of the conceptual validity criteria refer to the respective sections in the related OECD guidance documents:

Krebs: https://www.doi.org/10.1007/s00204-020-02802-6

PBK: https://www.doi.org/10.1787/d0de241f-en

QMRF: https://one.oecd.org/document/ENV/CBC/MONO(2023)32/ANN1/en/pdf

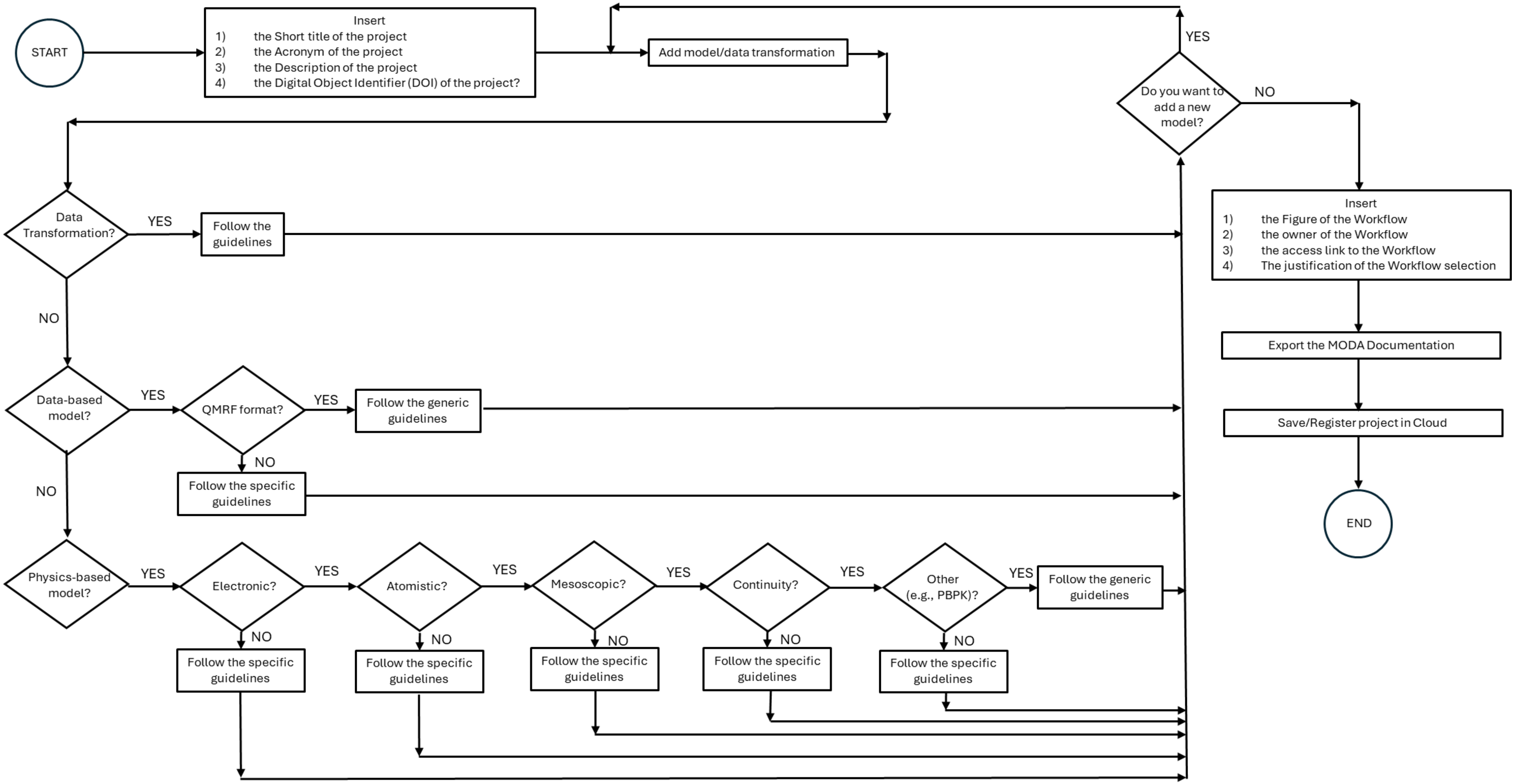

Figure S2: Flowchart of the Easy-MODA tool

References:

Lynch I & Afantitis A. (2024). WorldFAIR (D4.2) FAIRification of nanoinformatics tools and models recommendations. Zenodo. https://doi.org/10.5281/zenodo.10629631

Varsou D-D, Banerjee A, Roy J, Roy K, Savvas G, Sarimveis H, Wyrzykowska E, Balicki M, Puzyn T, Melagraki G, Lynch I, Afantitis A. (2024) The Round Robin approach applied to nanoinformatics: consensus prediction of nanomaterials zeta potential. Beilstein Arch. 2024, 202433. https://doi.org/10.3762/bxiv.2024.33.v1
