# Supplementary material for: Easy-MODA: Simplifying standardised registration of scientific simulation workflows through MODA template guidelines powered by the Enalos Cloud Platform
Source: Comput Struct Biotechnol J. 2024 Oct 18;25:256–68. doi: 10.1016/j.csbj.2024.10.018 (PMC11566491; doi:10.1016/j.csbj.2024.10.018)
Supplement: Supplementary file 2 — Supplementary material [file mmc2.pdf]

Moda powered by **Enalos Cloud Platform**

MODA for

Safety Assessment of Ag, TiO_2, and CuO nanoparticles

Simulated in project:

SafeNanoScope

OVERVIEW of the SIMULATION			
1	**USER CASE**	Safety Assessment of Ag, TiO_2, and CuO nanoparticles	
2	**CHAIN OF MODELS**	**Model 1**	construction of energy minimized NP<br>*Physics based model*
		**Model 2**	autoML<br>*Data Based model*
		**Data Transformation 3**	construction of geometrically constructed NP<br>*Data Transformation*
3	**PUBLICATION PEER - REVIEWING THE DATA**	DOI provided: **No**	
4	**ACCESS CONDITIONS**	Access type: **Free**<br>Owner of workflow: **NovaMechanics Ltd**<br>Workflow access link:<br>https://www.enaloscloud.novamechanics.com/sabydoma/safenanoscope/	
5	**WORKFLOW AND ITS RATIONALE**	Traditional (experimental) methods for assessing the nanoparticles (NPs) safety are time-consuming, expensive, and resource-intensive, and raise ethical concerns due to their reliance on animals. To address these challenges, we propose an in silico workflow that serves as an alternative or complementary approach to conventional hazard and risk assessment strategies, which incorporates state-of-the-art computational methodologies. In detail, an automated machine learning (autoML) scheme is developed employing dose-response toxicity data for silver (Ag), titanium dioxide (TiO_2), and copper oxide (CuO) NPs. This model is further enriched with atomistic descriptors using the ASCOT tool to capture the NPs' underlying structural properties. To overcome the issue of limited data availability, synthetic data generation techniques are used. These techniques help in broadening the dataset, thus improving the representation of different NP classes.	

Workflow picture

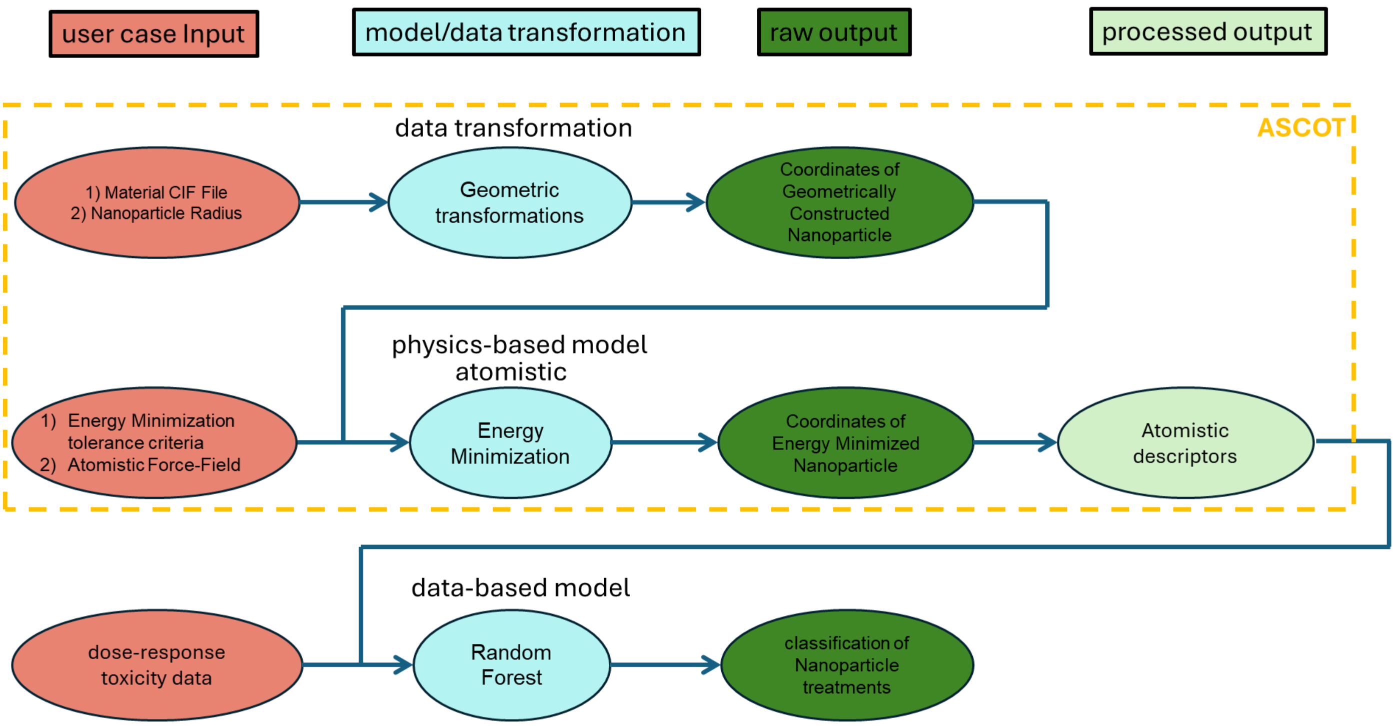

Each physics-based model used in this simulation is to be documented in four chapters:

1. Aspect of the User Case or system simulated with this model
2. Model: Please make sure the notions Physics Equation and Materials Relation are properly understood.
   - Tightly coupled models can be written up collectively in one set of four tables. To solve tightly coupled PE one matrix is set up and solved in one go.
   - For continuum models the PE is often the conservation equations coded up in bought software packages.
   - Often the MR is established by the modeller.
3. Computational aspects include also a documentation of how the user case specifications are translated into computer language.
4. Post processing documents how the raw output of one simulation is processed into input for the next simulation. This information given under 4.1 in the first model will be the same as the "simulated input" information under 2.4 for the next model. This is the essence of model inter-operability!
5. Pre-processing before the first model can be depicted in pink as it is considered to be part of the user-case.

Each data-based model in this simulation is to be documented in three chapters:

1. Aspect of the User Case or system simulated with this data-based model
2. Data-based Model
3. Computational detail of the datamining operation

MODA

Physics based Model

MODEL 1

construction of energy minimized NP

Aspect of the User Case/System to be Simulated		
**1.1**	**Aspect of the User Case to be simulated**	Digital Construction of energy minimized Ag, TiO_2 and CuO nanoparticles(NPs) having the diameters mentioned in the dataset needed to be enriched and calculation of their atomistic descriptors.
**1.2**	**Material**	Geometrically constructed Nanoparticles made by model "Construction of Geometrically Constructed NPs after using the following CIF files Ag (Fm-3 m space group, COD ID 1509146), TiO_2 (rutile P42/mnm, COD ID 1532819 and Anatase, I41/amd space group, COD ID 1010942), CuO (the space group C12/c1, tenorite, COD ID 1011148)
**1.3**	**Geometry**	Spherical initially and any shape that is created after the energy minimization procedure.

Aspect of the User Case/System to be Simulated		
**1.4**	**Time Lapse**	As an energy minimization procedure is used, if the converge criteria are satisfied, the procedure stops. The number of steps was 12 on average but this number depends on the size of the constructed NP.
**1.5**	**Manufacturing process or in-service conditions**	Energy minimization applied to geometrical constructed NPs with absence of periodic boundary conditions to simulate a NP in vaccum that it does not interact with itself.
**1.6**	**Publication on this data**	https://doi.org/10.1016/j.csbj.2024.03.011

Generic Physics Of The Model Equation			
**2.0**	**Model type and name**	Atomistic	
**2.1**	**Model entity**	Atoms	
**2.2**	**Model Physics/ Chemistry equation PE**	Equation	Conjugate gradient
		Physical quantities	Atomic forces<br>Coordinates<br>Potential energy
**2.3**	**Materials relations**	Relation	Liang et al - COMB3 FF (https://doi.org/10.1016/j.mser.2013.07.001)<br>OPENKIM Force-Fields:<br>EAM_Dynamo_AcklandTichyVitek_1987v2_Ag______MO_055919219575_000<br>Sim_LAMMPS_MEAM_ZhangTrinkle_2016_TiO______SM_513612626462_000<br>Sim_LAMMPS_IFF_PCFF_HeinzMishra LinEmami_2015Ver1v5_FccmetalsMineralsSolventsPolymers__SM_039297821658_000

Generic Physics Of The Model Equation			
		Physical quantities /descriptors for each MR	Interatomic distances. bonds, angles and dihedral angles.
2.4	**Simulated input**	Initial configuration file names<br>Ag(1509146.cif), TiO_2 (1532819.cif and 1010942.cif), CuO (1011148.cif) after its geometrical manipulation with ASCOT(https://www.enaloscloud.novamechanics.com/sabydoma/ascot/)	

Solver and Computational translation of the specifications			
3.1	**Numerical Solver**	Energy Minimization (Polak-Ribiere version of the conjugate gradient (CG) algorithm)	
3.2	**Software tool**	[opensource] LAMMPS integrated with Python scripts through ASCOT Interface	
3.3	**Time step**	no timestep	
3.4	**Computational Representation**	Physics Equation,<br>Material Relations,<br>Material	Energy minimization (Conjugate gradient)
3.5	**Computational boundary conditions**	no periodic boundary conditions	
3.6	**Additional Solver Parameters**	Long range(pppm, ewald)<br>FF cutoff = 1.2 nm<br>neighbor list frequency = 1<br>neighbor list cutoff = 1.4nm	

Post processing

The “raw output” calculated by the model consists per definition of values for the physics variable in the PE(s). This variable is already specified in 2.2 and this raw output will appear in your dark green circle in the workflow picture.

• to calculate values for physics variables for different entities of the next model. E.g. the output can be homogenised for larger volumes

- in the form of a MR for the next model
- into a Descriptor Rule that is the final output of the total simulation.

This processed output will appear in your light green circle in the workflow picture and also in 2.4 of the next model (if there is one).

The methodology (often including physics) used to do this post processing calculation is to be documented in 4.2.

**Solver and Computational translation of the specifications**		
**4.1**	**The processed output**	average potential energy per atom<br>average coordination number per atom<br>average neighbor parameter per atom<br>average hexatic order parameter per atom<br>for core and shell part of the Nanoparticle as well as the whole Nanoparticle
**4.2**	**Methodologies**	Average values
**4.3**	**Margin Of Error**	Standard deviation of the above properties has not been calculated

MODA

Data Based Model

MODEL 2

autoML

Aspect of the User Case/System to be Simulated		
**1.1**	**Aspect of the User Case to be simulated**	[Endpoint] The human hepatoma HepaRG cell line was treated with 89 NPs at 10 different concentrations, and 14 imaging endpoints were measured through a High Throughput Screening (HTS) – High Content Imaging (HCI) study to initially classify NP hazards and identify candidates for further toxicological assessment. The endpoints assessed included cell viability and mitochondrial health by measuring 9 features and the results of the HTS-HCI screening were normalised following the signal-to-noise ratio approach. A threshold of -3 for downward response and +3 for upward response was used, which corresponded to a 99% certainty that the cell behaviour was different from the untreated (negative) control value (cells treated only with medium).<br><br>[Comment on endpoint] The normalised values were depicted in a colour-coded heatmap, which reflected the extend of difference of the behaviour from the untreated control (red and blue colours for decreased or increased response, respectively) or indicated similar behaviour to the untreated control (green colour).<br><br>[Endpoint units] The results of the 9 toxicity features were summarised into a single endpoint (“overall”) class as follows: NP treatments were classified as “Low effect” if they had a similar response to the negative controls (green labels) in at least 5 measured features (73 NP treatments). Otherwise, NP treatments were classified as “High effect” (red and/or blue label, 37 treatments).<br><br>[Experimental protocol, Endpoint data quality and variability] Joossens, E., Macko, P., Palosaari, T. et al. A high throughput imaging database of toxicological effects of nanomaterials tested on HepaRG cells. Sci Data 6, 46 (2019). https://doi.org/10.1038/s41597-019-0053-2
**1.2**	**Material**	Ag (Fm-3 m space group, COD ID 1509146), TiO_2 (rutile P42/mnm, CODID 1532819 and Anatase, I41/amd space group, COD ID 1010942), CuO (the space group C12/c1, tenorite, COD ID 1011148)
**1.3**	**Geometry**	Spherical, faceted and rod-shaped (for more info see:

Aspect of the User Case/System to be Simulated		
		https://www.csbj.org/article/S2001-0370(24)00073-4/fulltext#secsect0030).<br><br>For non-spherical NPs, the following assumptions were made:<br>• Square/faceted NPs were treated as spheres with a diameter equal to the particles' equivalent sphere area diameter.<br>• Rod-shaped NPs were modelled as ellipsoid NPs. In this representation, the X-axis dimension equals the NPs longest diameter, and the Y and Z axes dimensions equal the NPs shortest diameter. Calculations for ellipsoid NPs involved three different rotation angles on the Z-axis (30°, 60° and 90°). The ellipsoid configuration yielding the minimum average potential energy of all atoms was selected as the most stable structure.
**1.4**	**Time Lapse**	The prediction of the effects class via the Enalos SafeNanoScope web-application is generated within seconds (depending on the size of the input data).
**1.5**	**Manufacturing process or in-service conditions**	[Description of the applicability domain of the model] To assess the applicability domain (AD) of the models we proposed a comprehensive approach where different AD assessment methods are combined to enhance the confidence of stakeholders in the produced predictions.<br>• In the bounding box (or range-based) method the interpolation space is considered the hyper-box defined by the range of minimum and maximum selected descriptor values.<br>• In the leverage method, the leverage values h -which are the diagonal elements of the Hat matrix- reflect the similarity of the validation or untested samples to the training set (distance from the training set's centroid) based on the descriptor values used in the model development. The limits of the AD are determined by the threshold leverage value h*. The prediction for a validation or untested NP is considered reliable if h<h*.<br>• The last approach is based on the similarity of the closest training NPs to the query NP. In detail, this approach starts by applying the k nearest neighbour (kNN) methodology to the query NPs to assess the NP's local region in the hyperspace. For each query NP the k closest training NPs are selected based on the Euclidean distances between them, calculated considering the selected descriptors. Next, the cosine similarity between the query NP and each of the k training NPs is calculated and it is compared to a predefined threshold (simk). If the similarity value of at least one of the k training NPs is below the threshold, the query NP is out of the AD limits and the prediction for this

Aspect of the User Case/System to be Simulated		
		NP is considered unreliable.<br><br>Note that as the models are built using data for Ag, TiO_2 and CuO NPs, ifthey are used to predict the behaviour of other types of NPs (extrapolation), the differences between them and the training NPs should be considered.<br><br>[Method used to assess the applicability domain] Three different AD methodologies were applied namely the bounding box, the leverage, andthe local similarity methods. The results of the three methodologies were summarised into a single weighted score value that classifies the overall predictions as “good”, “moderate”, and “poor”.<br><br>[Software name and version for applicability domain assessment] KNIMEAnalytics Platform v.5.1.2, Enalos+ nodes.<br><br>[Limits of applicability] • Bounding box: An untested NP is outside the AD, if at least one of its descriptor values is out of the range of the limitsof the corresponding descriptor defined by the training NPs.<br>• Leverage: h* = 0.440.<br>• Local similarity: k = 5 and simk = 0.8.<br><br>For any query NP it is possible to assess its reliability based on the ADusing a scoring system: =,+,+,<br>Where, , is the combined reliability score of the query NP, , , and arethe weighting factors of the bounding box, leverage and similarity AD methods respectively, and ,, ,, and , are binary variables indicating whether the query NP is inside (value of 1) or outside (value of 0) theAD limits of the model according to the three AD methods.<br>The proposed values for each weighting factor are: =0.2, =0.3, and =0.5.Finally, the overall reliability of the prediction is proposed to be defined as follows: <0.5 =0.5 >0.5<br><br>Note that as the models are built using data for Ag, TiO_2 and CuO NPs, if they are used to predict the behaviour of other types of NPs (extrapolation), the differences between them and the training NPs should be considered.
**1.6**	**Publication on this data**	https://doi.org/10.1016/j.csbj.2024.03.020

The Data-based Model		
**2.0**	**Equation type and name**	[Explicit algorithm] Machine learning/Random Forest: Random forests are supervised ensemble learning algorithms that utilize bagging (bootstrap aggregating) and feature randomness to construct a multitude of decision trees. The selection of the random forest was a result of optimization via an autoML scheme.<br><br>Optimised Random Forest parameters: maxLevels = 4, minNodesize = 20, nrModels = 200.<br><br>Information on Random Forests: Tin Kam Ho, "Random decision forests," Proceedings of 3rd International Conference on Document Analysis and Recognition, Montreal, QC, Canada, 1995, pp. 278-282 vol.1, doi: 10.1109/ICDAR.1995.598994.
**2.1**	**Database and type**	[Availability of the training set] The training set is available as a supporting information file of the publication: Varsou et al. In Silico Assessment of Nanoparticle Toxicity Powered by the Enalos Cloud Platform: Integrating Automated Machine Learning and Synthetic Data for Enhanced Nanosafety Evaluation, Computational and Structural Biotechnology Journal, 2024. and at the NanoPharos DB (https://db.nanopharos.eu/Queries/Datasets.zul?datasetID=16).<br><br>[Available information for the training set] Nanomaterials dataset including physicochemical characterisation of the NPs and data on their in vitro toxicity to HepRG cells.<br><br>[Data for each descriptor variable for the training set] The training set is available as a supporting information file of the publication: Varsou et al. In Silico Assessment of Nanoparticle Toxicity Powered by the Enalos Cloud Platform: Integrating Automated Machine Learning and Synthetic Data for Enhanced Nanosafety Evaluation, Computational and Structural Biotechnology Journal, 2024. and at the NanoPharos DB (https://db.nanopharos.eu/Queries/Datasets.zul?datasetID=16).

The Data-based Model

[Data for the dependent variable (response) for the training set] The training set is available as a supporting information file of the publication: Varsou et al. In Silico Assessment of Nanoparticle Toxicity Powered by the Enalos Cloud Platform: Integrating Automated Machine Learning and Synthetic Data for Enhanced Nanosafety Evaluation, Computational and Structural Biotechnology Journal, 2024. and at the NanoPharos DB (https://db.nanopharos.eu/Queries/Datasets.zul?datasetID=16).

The training set comprises of 57 NP treatments randomly selected from the pool of the original NP treatments using stratified sampling. The training NP treatments are categorised as follows: 38 “Low effect” and 19 “High effect”.

[Pre-processing of data before modelling] Considering the class imbalance (66% “Low effect” vs. 34% “High effect” treatments) in the training set, the minority class (“High effect”) was oversampled to ensure that the number of treatments for each endpoint class is approximately equal, by employing the ADASYN methodology using k=5 neighbours. After oversampling the training set reached the 75 NP treatments (also available with the rest of the dataset). The training data were fed to a low variance filter (low variance threshold equal to 0.2) and to a Spearman’s rank correlation coefficient filter (correlation threshold equal to 0.95), to remove non-essential descriptors. Variable selection was performed by calculating the information gain (see §2.2) of all remaining descriptors and descriptors with zero information gain score are excluded from the modelling, as they are not considered critical for establishing a predictive relationship. After the above filtering steps, the training set is reviewed for possible duplicate treatments that occurred from the filtering of descriptors. Finally, the selected training descriptors are normalised using the z-score (Gaussian) method, to ensure their equal contribution to the analysis. The same normalisation functions are later applied to the test and blind sets.

[Availability of the external validation set] The blind set is available as supporting information file of the publication: Varsou et al. In Silico Assessment of Nanoparticle Toxicity Powered by the Enalos Cloud Platform: Integrating Automated Machine Learning and Synthetic Data for Enhanced Nanosafety Evaluation, Computational and Structural Biotechnology Journal, 2024. and at the NanoPharos DB (https://db.nanopharos.eu/Queries/Datasets.zul?datasetID=16).

[Available information for the external validation set] Nanomaterials dataset

The Data-based Model

<table>
<tr><td></td><td></td><td colspan="2">[Data for each descriptor variable for the external validation set] The blind set is available as supporting information file of the publication: Varsou et al. In Silico Assessment of Nanoparticle Toxicity Powered by the Enalos Cloud Platform: Integrating Automated Machine Learning and Synthetic Data for Enhanced Nanosafety Evaluation, Computational and Structural Biotechnology Journal, 2024. and at the NanoPharos DB (https://db.nanopharos.eu/Queries/Datasets.zul?datasetID=16).

[Data for the dependent variable for the external validation set] The blind set is available as supporting information file of the publication: Varsou et al. In Silico Assessment of Nanoparticle Toxicity Powered by the Enalos Cloud Platform: Integrating Automated Machine Learning and Synthetic Data for Enhanced Nanosafety Evaluation, Computational and Structural Biotechnology Journal, 2024. and at the NanoPharos DB (https://db.nanopharos.eu/Queries/Datasets.zul?datasetID=16).

[Other information about the external validation set] Blind set with 33 NP treatments appended.

[Experimental design of test set] Random-stratified selection of NP treatments before modelling (30% of the original set).</td></tr>
<tr><td rowspan="2">2.2</td><td rowspan="2">Equation</td><td>Hypothesis</td><td>[Type of model] Non-linear model</td></tr>
<tr><td>Physical quantities</td><td>[Descriptors in the model]
Selected descriptors | Information gain score
Concentration of NPs in g/mL | 0.482
AD45: The average difference of the CNP (3Ang) between core and shell atoms| 0.145
AD27: The average difference of the coordination parameter (5Ang) between core and shell atoms | 0.145
AD22: The average difference of the coordination parameter (4Ang) between core and shell atoms | 0.145
AD17: The average difference of the coordination parameter (3Ang) between core and shell atoms | 0.145
AD16: The average coordination parameter (3Ang) of the shell atoms | 0.169
AD14: The average coordination parameter (3Ang) of all atoms | 0.145
AD9: The average coordination parameter of all atoms | 0.145
AD7: The average difference of the potential energy between</td></tr>
</table>

The Data-based Model			
			core and shell atoms in eV | 0.150<br>AD3: Log10 of all atoms in the surface| 0.145<br>AD1: Log10 of all atoms in the NP | 0.145<br><br>[Descriptor selection] From the initial pool of descriptors (53 in total), 33 were filtered out using missing values, low variance and correlation filtering (see §2.1). The information gain of all remaining descriptors (20) is calculated and descriptors with zero information gain score are excluded from the modelling, as they are not considered critical for establishing a predictive relationship. Finally, 11 descriptors were selected.<br><br>[Algorithm and descriptor generation] Atomistic simulations. To perform the simulations and acquire the computational descriptors, the size, the shape, and the phase of the NPs were needed (see also §1.3).<br><br>[Software name and version for descriptor and algorithm generation] • ASCOT: A Web Tool for the Digital Reconstruction of Energy Minimized Ag, CuO, and TiO Spherical Nanoparticles and Calculation of their Atomistic Descriptors Powered by Enalos SABYDOMA Cloud Platform, https://www.enaloscloud.novamechanics.com/sabydoma/ascot/<br>• For ellipsoid NPs see the extension of the ASCOT tool, NanoConstruct: Nanoparticle Construction Tool Powered by Enalos RiskGONE Cloud Platform, http://enaloscloud.novamechanics.com/riskgone/nanoconstruct/<br><br>[Chemicals/ Descriptors ratio] 75 training NP treatments (after oversampling)/ 11 selected descriptors.

Computational detail of datamining operation		
**3.1**	**Numerical Operations**	The collected data (NP characteristics, treatments and classification, enriched with atomistic descriptors) is investigated in silico, by developing ML models that could predict the adverse effects of NPs from their computational descriptors. It is noted that the modelling is performed considering the OECD principles for the validation of QSAR models (a defined endpoint, an unambiguous algorithm, a defined domain of applicability, appropriate measures of goodness-of-fit, robustness and predictivity, and a mechanistic interpretation, if possible). To begin with, a subset of the data is randomly selected using a stratified sampling technique and excluded from the model development process, to later serve as a blind set for validation purposes (see §2.1). The remaining data is fed to the autoML workflow and is split again into training and test sets (see §2.1); the training set is used for model development whereas the test set is used to select the best performing model inside an autoML scheme. The training data is oversampled to balance the relative frequency of the two endpoint classes ("low effect" and "high effect") (see §2.1). Later, data is filtered and fed into the core of the autoML modelling (see §2.1) where the best performing model among seven tested and optimised algorithms is selected as the final model. The best performing model (see §2.0) is applied on the blind set to assess the model's performance in real conditions (see §3.3). The applicability domain of the model is assessed using three different techniques (see §1.5) and the model is released as a web-service through the Enalos Cloud Platform.
**3.2**	**Software tool**	The KNIME (Konstanz Information Miner) Analytics Platform was used to perform data analysis (including the synthetic data generation and filtering), modelling and validation, as well as defining the applicability domain. For this purpose, different extensions were integrated into the KNIME workflow such as the Enalos+ nodes, the R programming language, the Palladian nodes and the AutoML component. The AutoML component was customised to incorporate the synthetic data generation, the filtering, and the variable selection steps of the analysis prior to modelling. Later, the deployment of the model as a user-friendly application was made via the Isalos Analytics Platform which permits deployment and sharing of ML models as web-services for straightforward access by the broader community, via the Enalos Cloud Platform.
**3.3**	**Margin Of Error**	[Statistics for goodness-of-fit] Statistics on the internal test set.

Computational detail of datamining operation		
		Accuracy: 0.95<br>MCC: 0.90<br>Recall (sensitivity): 1.00<br>Specificity: 0.92<br>Precision: 0.88<br>Cohen's Kappa: 0.89<br><br>[Robustness – Statistics obtained by leave-one-out cross-validation] (training set):<br>Accuracy: 0.88<br>MCC: 0.76<br>Recall (sensitivity): 0.92<br>Specificity: 0.84<br>Precision: 0.85<br>Cohen's Kappa: 0.76<br><br>[Robustness – Statistics obtained by five-fold cross-validation] (random selection, training set):<br>Accuracy: 0.85<br>MCC: 0.73<br>Recall (sensitivity): 0.97<br>Specificity: 0.74<br>Precision: 0.78<br>Cohen's Kappa: 0.71<br><br>[Robustness – Statistics obtained by Y-scrambling]<br>Randomisation | Accuracy | MCC<br>1 | 0.25 | -0.42<br>2 | 0.45 | -0.10<br>3 | 0.35 | -0.21<br>4 | 0.65 | 0.31<br>5 | 0.60 | 0.12<br>6 | 0.50 | 0.10<br>7 | 0.60 | 0.32<br>8 | 0.40 | -0.24<br>9 | 0.55 | 0.10<br>10 | 0.60 | 0.18<br><br>[Predictivity – Statistics obtained by external validation]<br>Accuracy: 0.88

Computational detail of datamining operation		
		MCC: 0.75<br>Recall (sensitivity): 0.91<br>Specificity: 0.86<br>Precision: 0.77<br>Cohen's Kappa: 0.74<br><br>[Predictivity – Assessment of the external validation set]<br>The blind set is sufficiently large and as a stratified sampling technique was applied for its selection, it is ensured that the class distribution (“Low effect”/ “High effect”) in the sets is representative of the original data. It was also assessed whether the blind set treatments were inside the AD limits and the results can be found in the supporting information file of the publication: Varsou et al. In Silico Assessment of Nanoparticle Toxicity Powered by the Enalos Cloud Platform: Integrating Automated Machine Learning and Synthetic Data for Enhanced Nanosafety Evaluation, Computational and Structural Biotechnology Journal, 2024.<br><br>[Comments on the external validation of the model] It is noted that considering that the modelling was performed inside an automated-ML scheme, the final selection of the model was based on the performance on an internal test set (statistics presented in above). The blind set was used as an external validation set. For more information, please refer to the relevant publication: Varsou et al. In Silico Assessment of Nanoparticle Toxicity Powered by the Enalos Cloud Platform: Integrating Automated Machine Learning and Synthetic Data for Enhanced Nanosafety Evaluation, Computational and Structural Biotechnology Journal, 2024.

MODA

Data Transformation

Data Transformation 3

construction of geometrically constructed NP

Aspect of the User Case		
**1.1**	**Aspect of the User Case to be simulated**	Digital geometrical Construction of Ag, TiO_2 and CuO nanoparticles (NPs)having the diameters mentioned in the dataset needed to be enriched and calculation of their atomistic descriptors.
**1.2**	**Material**	Ag (Fm-3 m space group, COD ID 1509146), TiO_2 (rutile P42/mnm, CODID 1532819 and Anatase, I41/amd space group, COD ID 1010942), CuO (the space group C12/c1, tenorite, COD ID 1011148)
**1.3**	**Geometry**	Spherical
**1.4**	**Time Lapse**	As a data-transformation method, there is not simulation time. The real time needed for the NP to be built is approximately 3 seconds.
**1.5**	**Manufacturing process or in-service conditions**	The algorithm that was used for this model is mentioned in the ASCOT web application scientific publication (https://doi.org/10.1016/j.csbj.2024.03.011). It includes first the removement of atoms from the box that lie outside the sphere's diameter, next the species with a greater proportion of atoms relative to the total number indicated by its chemical formula are identified and finally the exact number of atoms to remove from each species to maintain the correct stoichiometry is calculated, the atoms located within an inner sphere, which has a radius 0.02Å smaller than the desired NP are found and from them the atoms in the box that fall within a shell of thickness 0.02Å (i.e., outside the inner sphere) and belonging to the excess species are removed until the stoichiometry aligns with the chemical formula.
**1.6**	**Publication on this data**	https://doi.org/10.1016/j.csbj.2024.03.011

Data Transformation			
**2.0**	**Equation type and name**	Geometrical manipulations were used (e.g. unit cell replication) and an algorithmic procedure mentioned in detail in the section "MANUFACTURING PROCESS OR IN-SERVICE CONDITIONS"	
**2.1**	**Database and type**	Initial configuration file names Ag(1509146.cif), TiO_2 (1532819.cif and 1010942.cif), CuO (1011148.cif)	
**2.2**	**Equation**	Hypothesis	Geometrical manipulations were used (e.g. unit cell replication) and an algorithmic procedure mentioned in detail in the section "MANUFACTURING PROCESS OR IN-SERVICE CONDITIONS"
		Physical quantities	Coordinates of atoms of the NP

Computational detail		
**3.1**	**Numerical Operations**	Geometrical manipulations were used (e.g. unit cell replication) and an algorithmic procedure mentioned in detail in the section "MANUFACTURING PROCESS OR IN-SERVICE CONDITIONS"
**3.2**	**Software tool**	ASCOT web application (https://www.enaloscloud.novamechanics.com/sabydoma/ascot/)
**3.3**	**Margin Of Error**	Due to strictly mathematical operations used during the geometrical construction of NP there is no margin of error except the errors may have been inserted due to errors of the inserted input files.
