# Supplementary material for: Easy-MODA: Simplifying standardised registration of scientific simulation workflows through MODA template guidelines powered by the Enalos Cloud Platform
Source: Comput Struct Biotechnol J. 2024 Oct 18;25:256–68. doi: 10.1016/j.csbj.2024.10.018 (PMC11566491; doi:10.1016/j.csbj.2024.10.018)
Supplement: Supplementary file 3 — Supplementary material [file mmc3.pdf]

# Easy-MODA

*"Simplifying Standardized Registration of Scientific Simulation Workflows through MODA Template Guidelines"*

**User Manual**

email: info@novamechanics.com

INSIGHT

WorldFAIR

*Easy-MODA web application is powered by Enalos Cloud Platform and is accessible through the link https://www.enaloscloud.novamechanics.com/insight/moda/

**Easy-MODA web application aspires to facilitate the Modelling Data (MODA) registration according to the guidelines that have been proposed by the European Materials Modelling Council (EMMC) and are available in the following links: a) https://emmc.info/moda-workflow-templates/ and b) https://emmc.eu/wp-content/uploads/2021/05/EMMC_IntWorkshop_Vienna2017_MODA_Talk.pdf

The user can access **Easy-MODA** through the link https://www.enaloscloud.novamechanics.com/insight/moda/ or by visiting the Enalos Cloud Platform and searching for the tool.

By clicking the above link, the Graphical User Interface (GUI) of **Easy-MODA** will appear (see the Figure on the right).

The user can select among three view modes (light, green and dark).

There are placeholders that guide the user through the information that is required to be typed into the GUI.

The user can add a model by clicking the **"Add Model/Data Transformation"** button (see next slide for more details) and insert a workflow picture (see later slides).

Next, the project's manual document is downloaded by clicking on the **"Create Document"** button.

Finally, the user can save the inserted data to continue later by clicking on the **"Export"** button, and upload it later by clicking on the **"Load Version"** button.

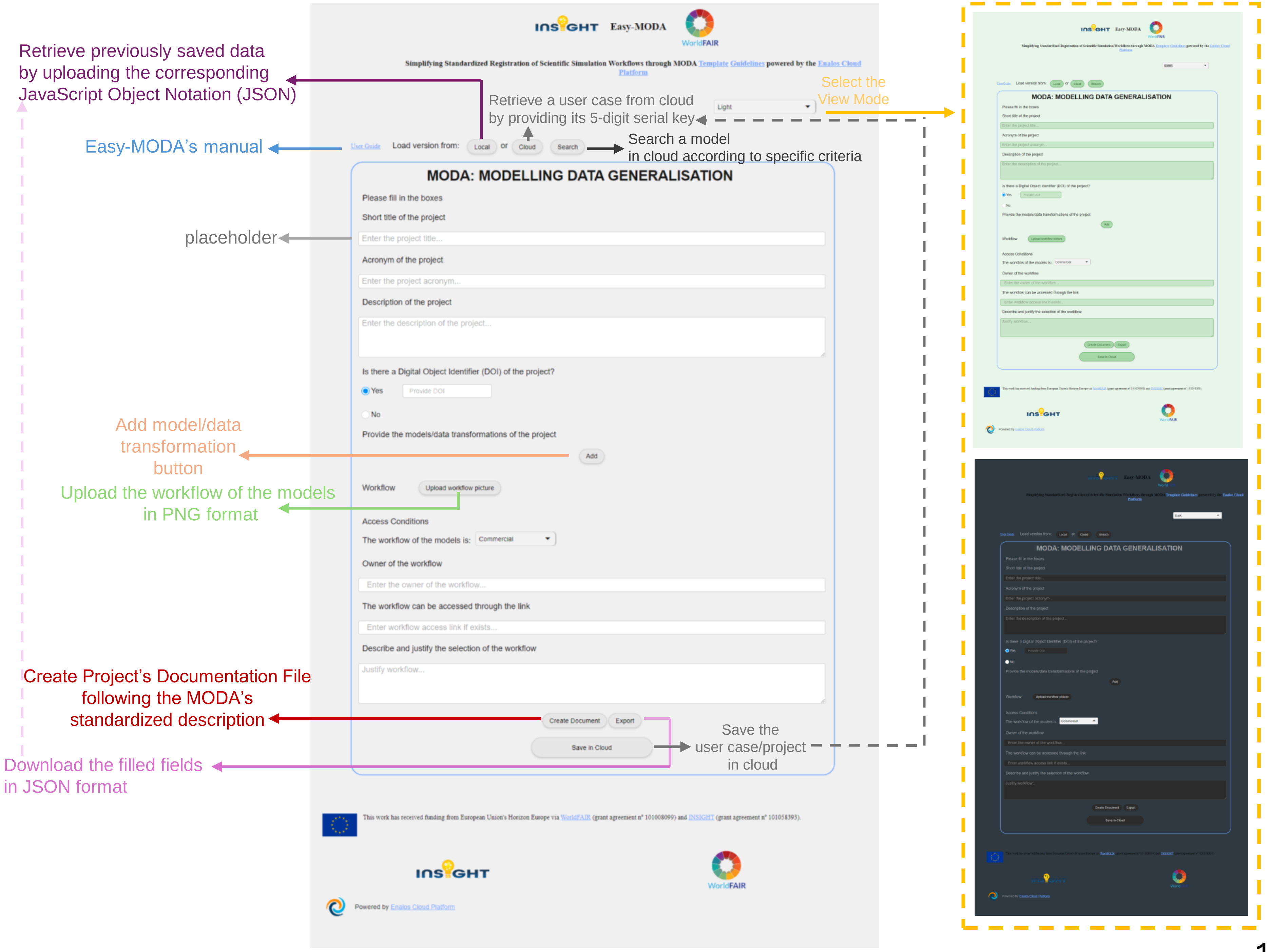

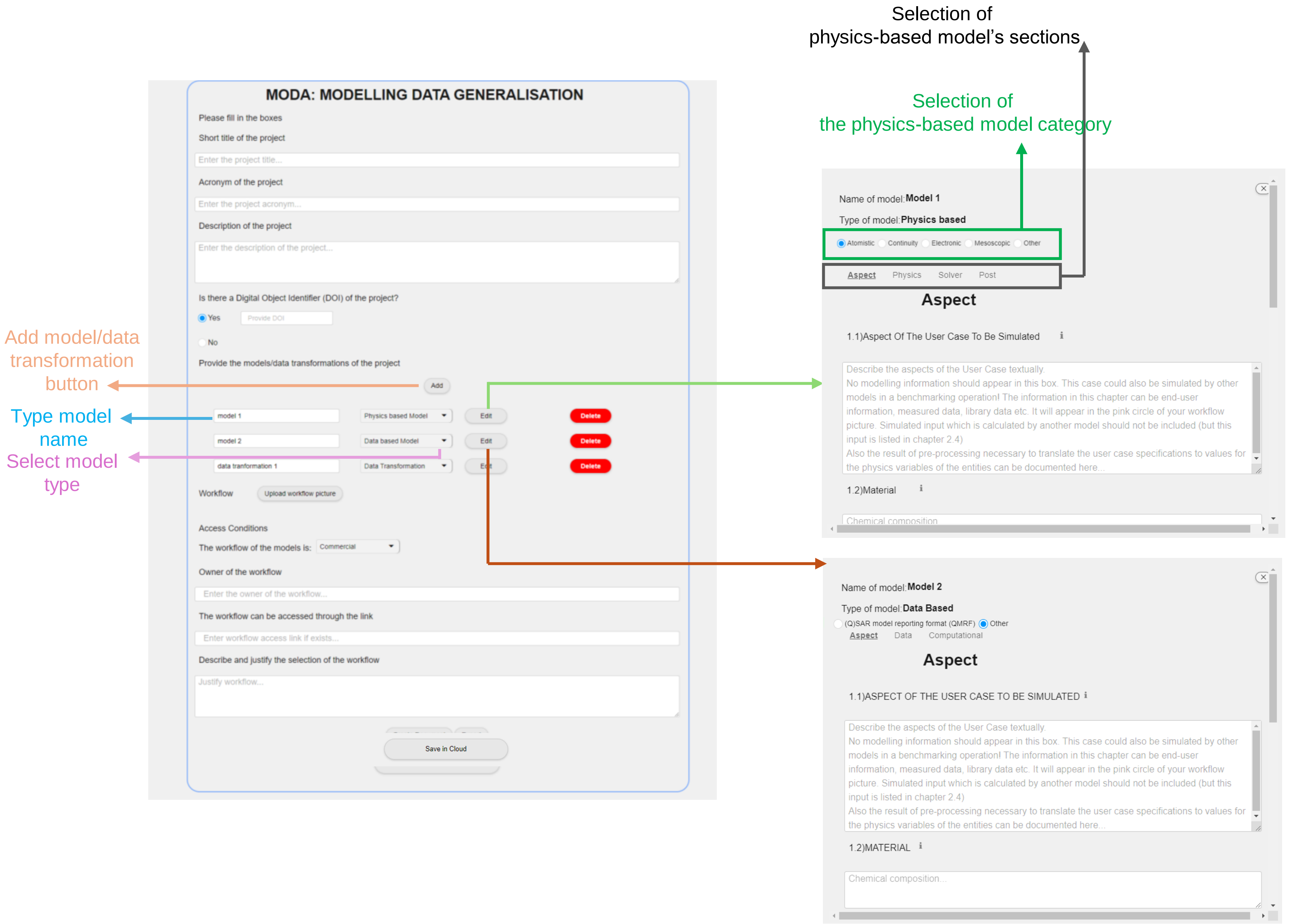


By clicking on the **"Add Model/Data Transformation"** button (see the Figure on the left), a new line is created in the Graphical User Interface, where the user types the name of the model, select the model's type (i.e., physics-based or data-driven) and clicks on the "Edit Model" button so that another windows opens where the user can fill in the details of the model.

The user shall select the category of the physics-based model (i.e., Atomistic, Continuity, Electronic, Mesoscopic) which leads to an automated filling of the rest of the fields of the window.

The physics-based models template consists of four sections (i.e., Aspect, Physics, Solver, Post) that need to be filled to follow the MODA guidelines.

The data-based models template consists of only three sections (i.e., Aspect, Data, Computational).

We elaborate more on how to fill the fields in these windows in the next slides.

The user can get more information about how the fields have been filled for models developed in other projects by moving the mouse cursor over the **"i"** button.

If the user later selects another physics-based model category the information that has already been inserted into the fields is erased to assure that that the typed information is compatible with the selected model category.

The user can click on the tabs **"Aspect"**, **"Physics"**, **"Solver"** and **"Post"** to fill in all of the fields needed to describe the model and enhance its re-usability by others.

The **"Scroll down"** bar can be used to access each field of the tab.

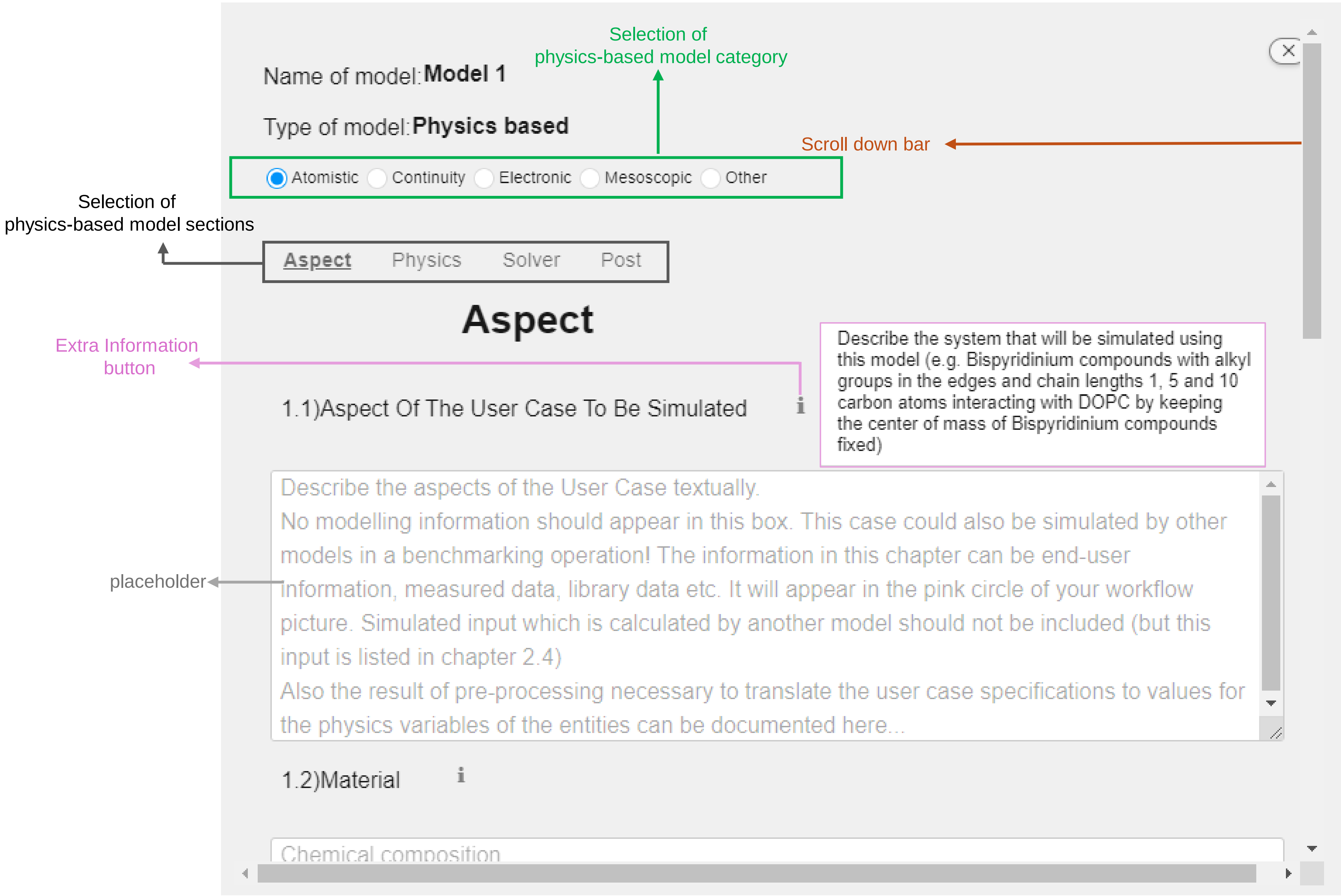

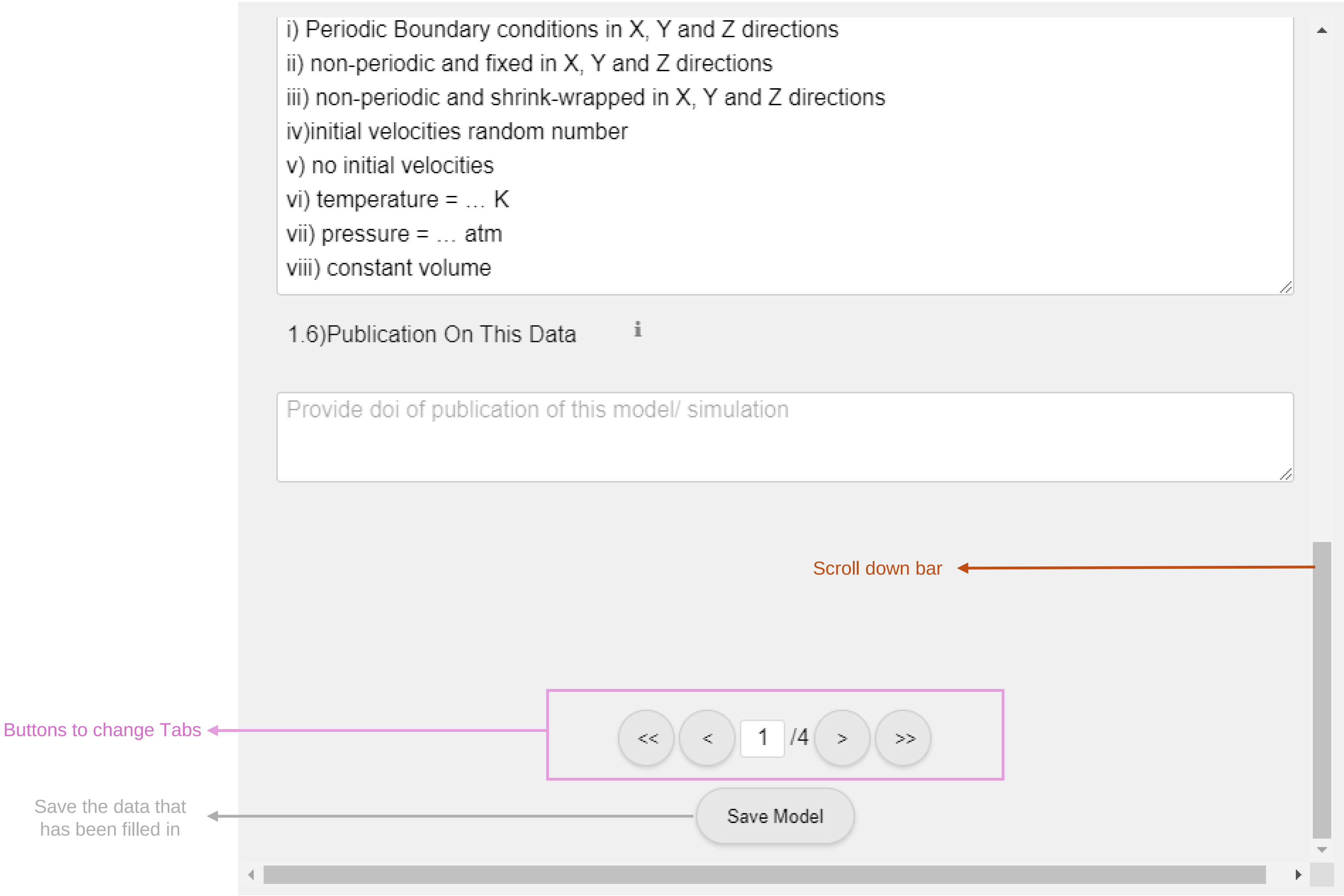


The user can also change tabs by selecting the arrow buttons at the bottom of the model's window.

If the user wants to save the entries (e.g., to complete further sections at a later time), then the **"Save Model"** button shall be used, otherwise the information will get lost if the user closes the window.

The user should also click on the **Export Data** button on the main GUI (see slide 1) to download the inserted information for the project and its models in order to save its entries and to be able to reload it.

By clicking on the **"Upload Workflow Picture"** button (see the Figure on the right), the user can upload the workflow image which shall create manually based on the information inserted in each model.

The information in the fields *"Material"*, *"Geometry"* of the **"Aspect"** tab and *"time step"* and *"computational boundary conditions"* in the **"Solver"** tab are the text of the red boxes of the workflow (see bottom right of the figure).

The information inserted in the *"numerical solver"* field of the **"Solver"** tab is shown in the light blue boxes of the workflow (see bottom right of the figure).

The field of "physical quantities" of the "**Physics**" tab is the raw output of the workflow (see the green boxes in the bottom right of the figure).

The field of "processed output" of the **"Post"** tab is the final (processed) output from the model which is written in the light green box of the workflow (see bottom right of figure).

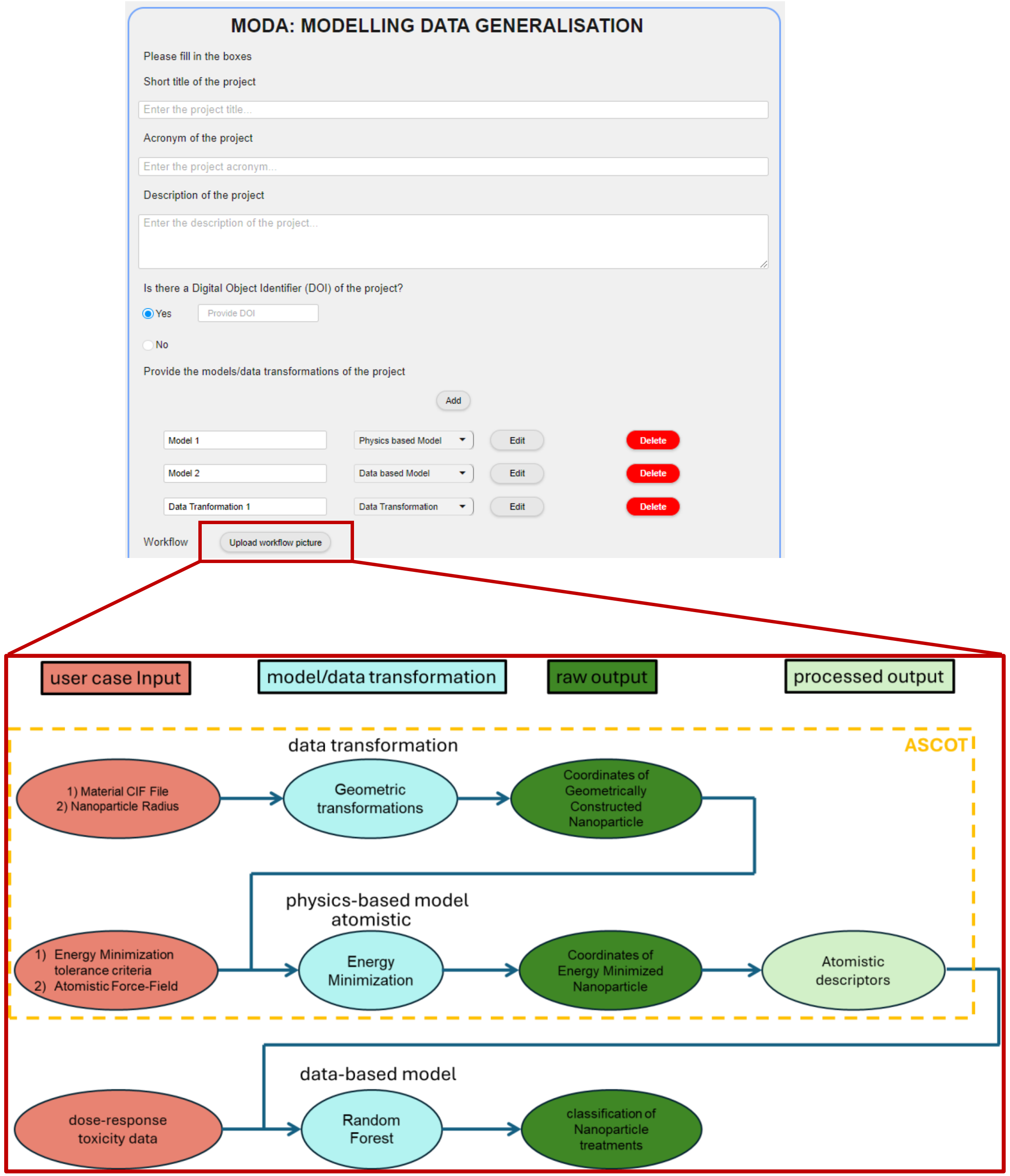

By clicking on the **"Upload Workflow Picture"** button (see the Figure on the right), the user can upload the workflow image which shall create manually based on the information inserted in each model.

The information in the fields *"Material"*, *"Geometry"* of the **"Aspect"** tab and *"time step"* and *"computational boundary conditions"* in the **"Solver"** tab are the text of the red boxes of the workflow (see bottom right of the figure).

The information inserted in the *"numerical solver"* field of the **"Solver"** tab is shown in the light blue boxes of the workflow (see bottom right of the figure).

The field of "physical quantities" of the "**Physics**" tab is the raw output of the workflow (see the green boxes in the bottom right of the figure).

The field of "processed output" of the **"Post"** tab is the final (processed) output from the model which is written in the light green box of the workflow (see bottom right of figure).

| user case Input | model | raw output | processed output |
|---|---|---|---|
| Physics-based<br>a) **"Aspect" tab**<br>"Material",<br>"Geometry"<br>b) **"Solver" tab**<br>"time step" ,<br>"computational boundary conditions"<br><br>Data-based<br>a) **"Data" tab**<br>"Database and type" | Physics-based<br>a) **"Solver" tab**<br>"numerical solver"<br><br>Data-based<br>a) **"Data" tab**<br>"Equation Type and Name"<br>b) **"Computational" tab**<br>"numerical operations" | Physics-based<br>a) **"Physics" tab**<br>"physical quantities"<br><br>Data-based<br>a) **"Data" tab**<br>"physical quantities" | Physics-based<br>a) **"Post" tab**<br>"processed output" |

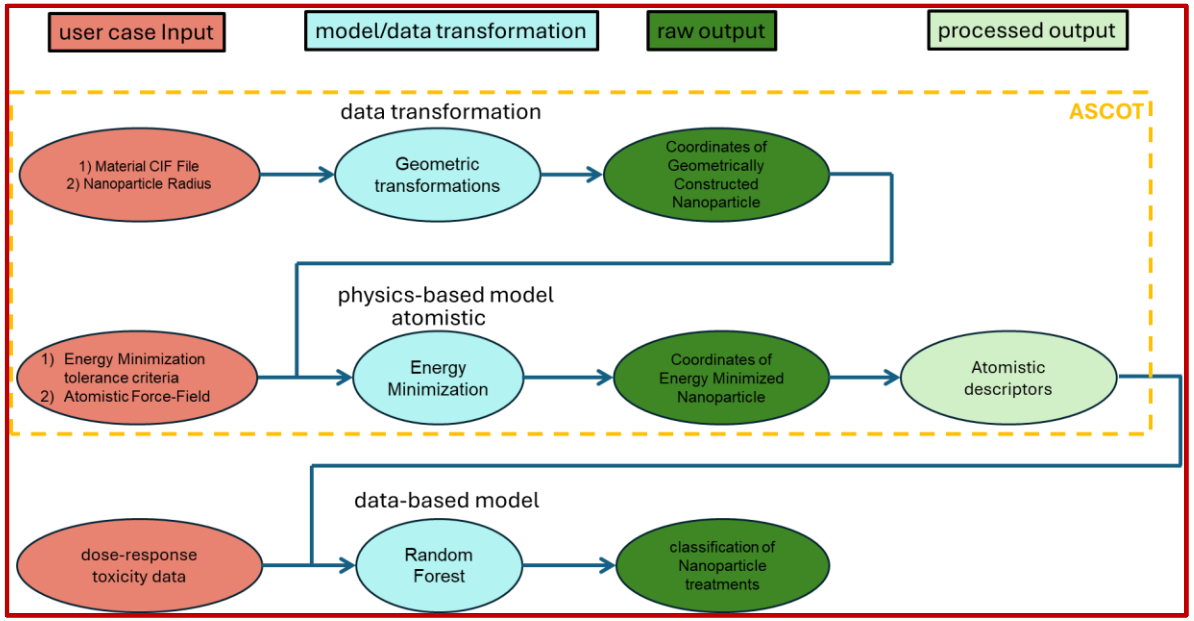

By clicking on the **“Upload Workflow Picture”** button (see the Figure on the right), the user can upload the workflow image which shall create manually based on the information inserted in each model.

The information in the fields *“Material”*, *“Geometry”* of the **“Aspect”** tab and *“time step”* and *“computational boundary conditions”* in the **“Solver”** tab are the text of the red boxes of the workflow (see bottom right of the figure).

The information inserted in the *“numerical solver”* field of the **“Solver”** tab is shown in the light blue boxes of the workflow (see bottom right of the figure).

The field of “physical quantities” of the “**Physics**” tab is the raw output of the workflow (see the green boxes in the bottom right of the figure).

The field of “processed output” of the **“Post”** tab is the final (processed) output from the model which is written in the light green box of the workflow (see bottom right of figure).

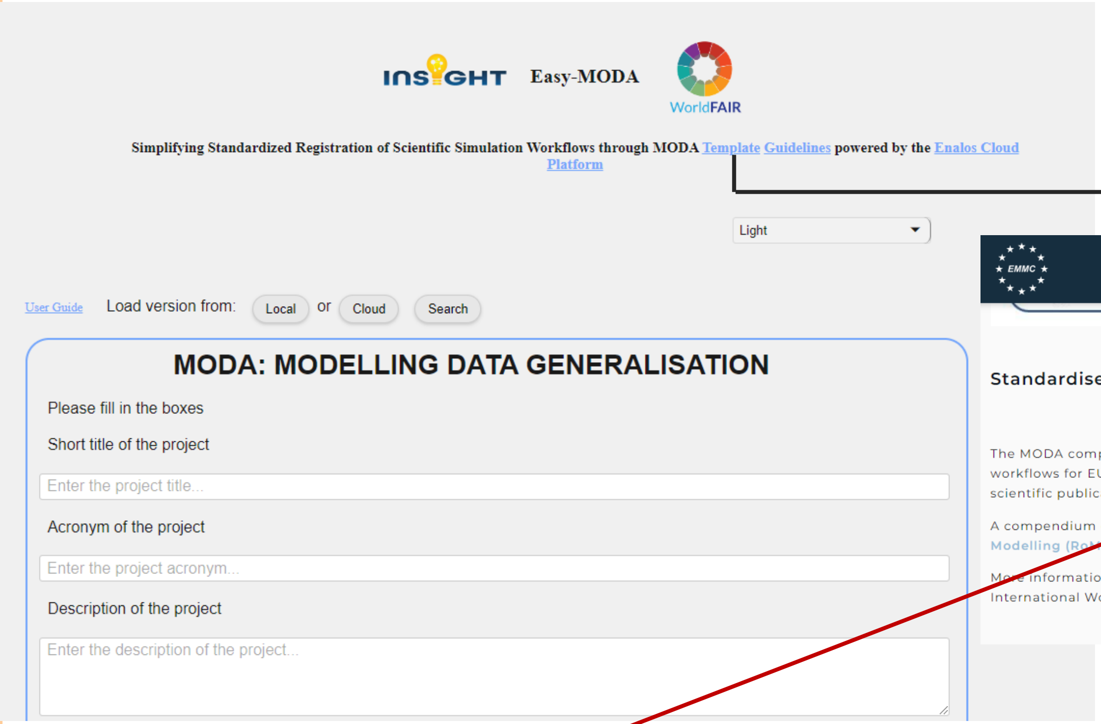


https://emmc.eu/moda/

By clicking this link, the EMMC website appears and Workflow Templates can be downloaded

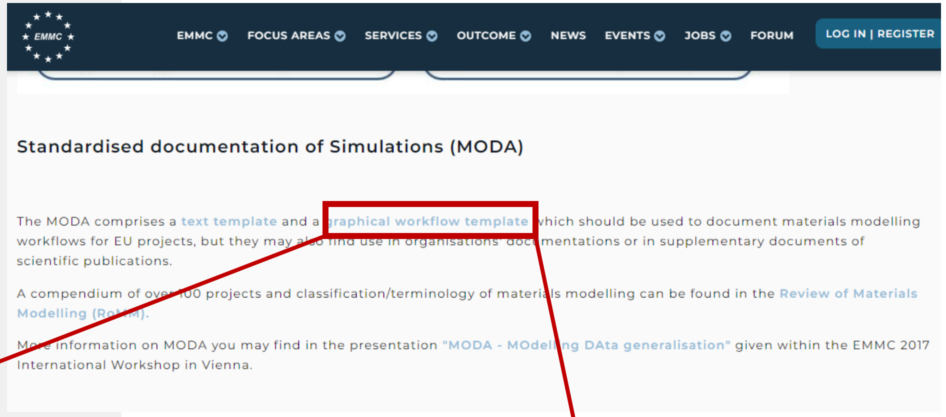


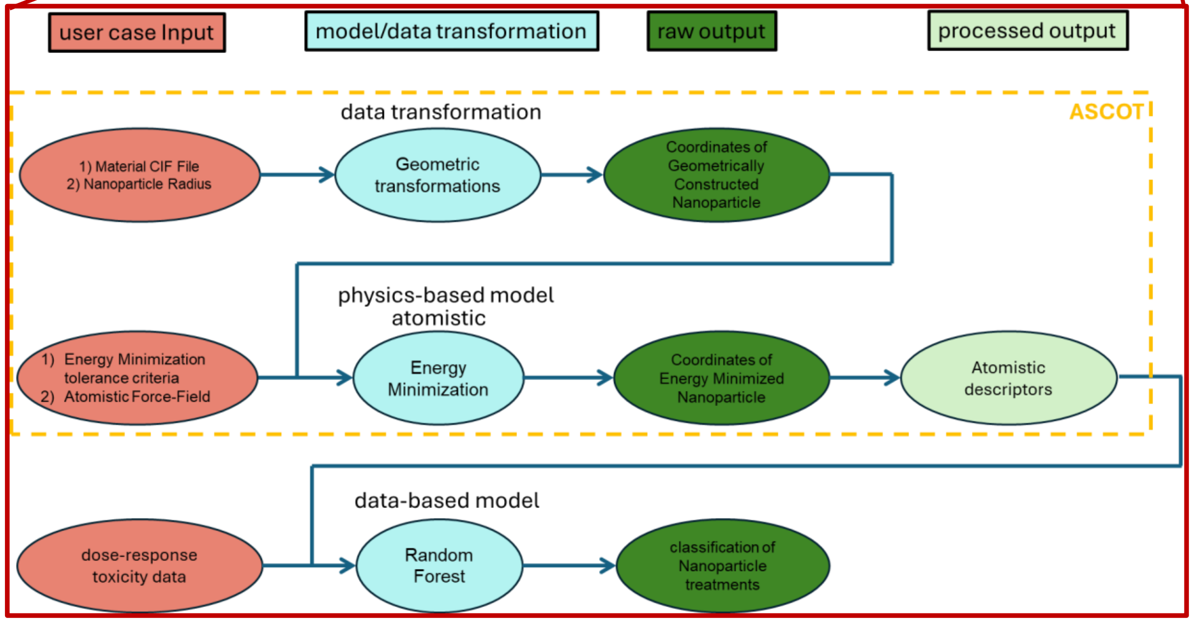

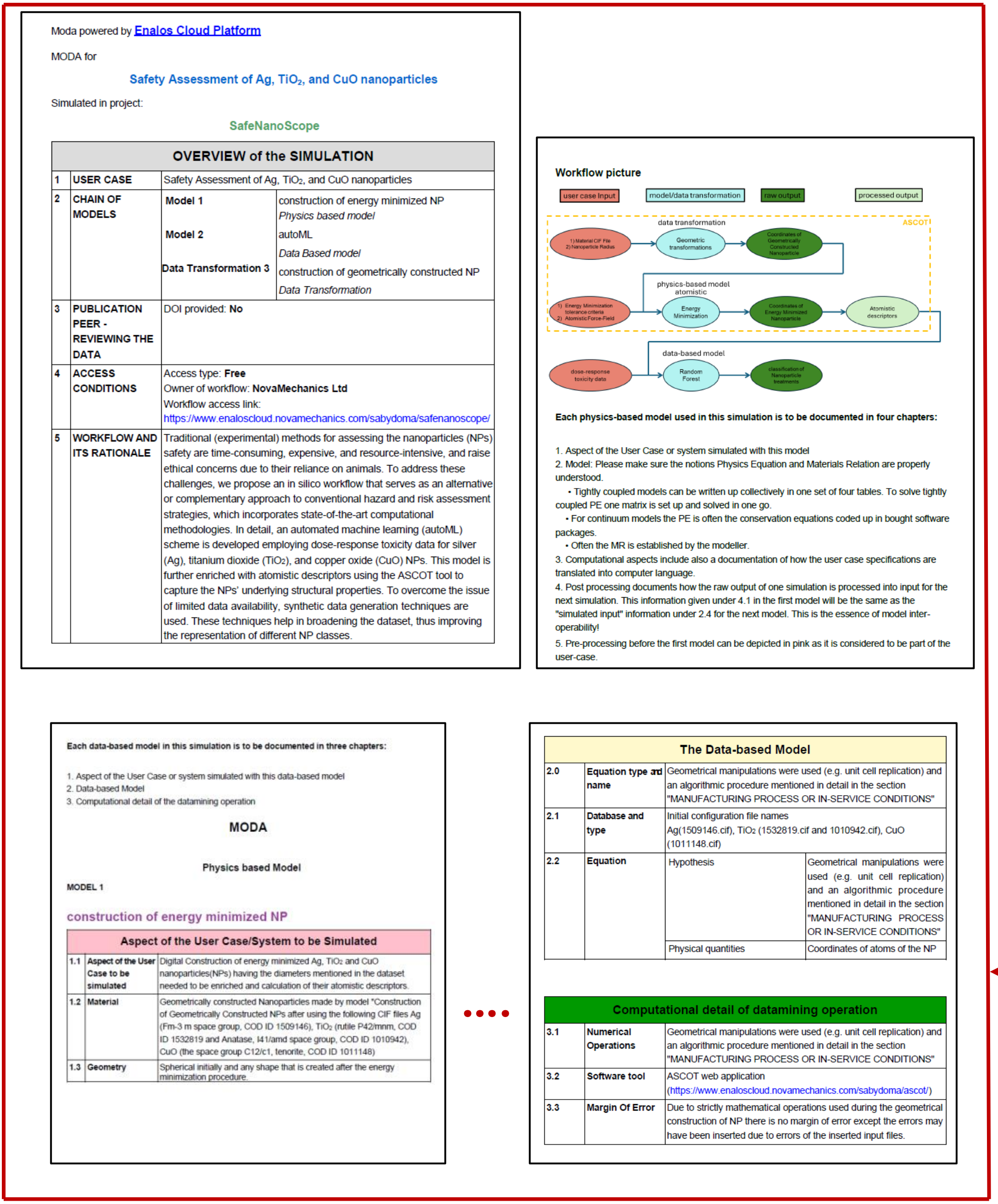


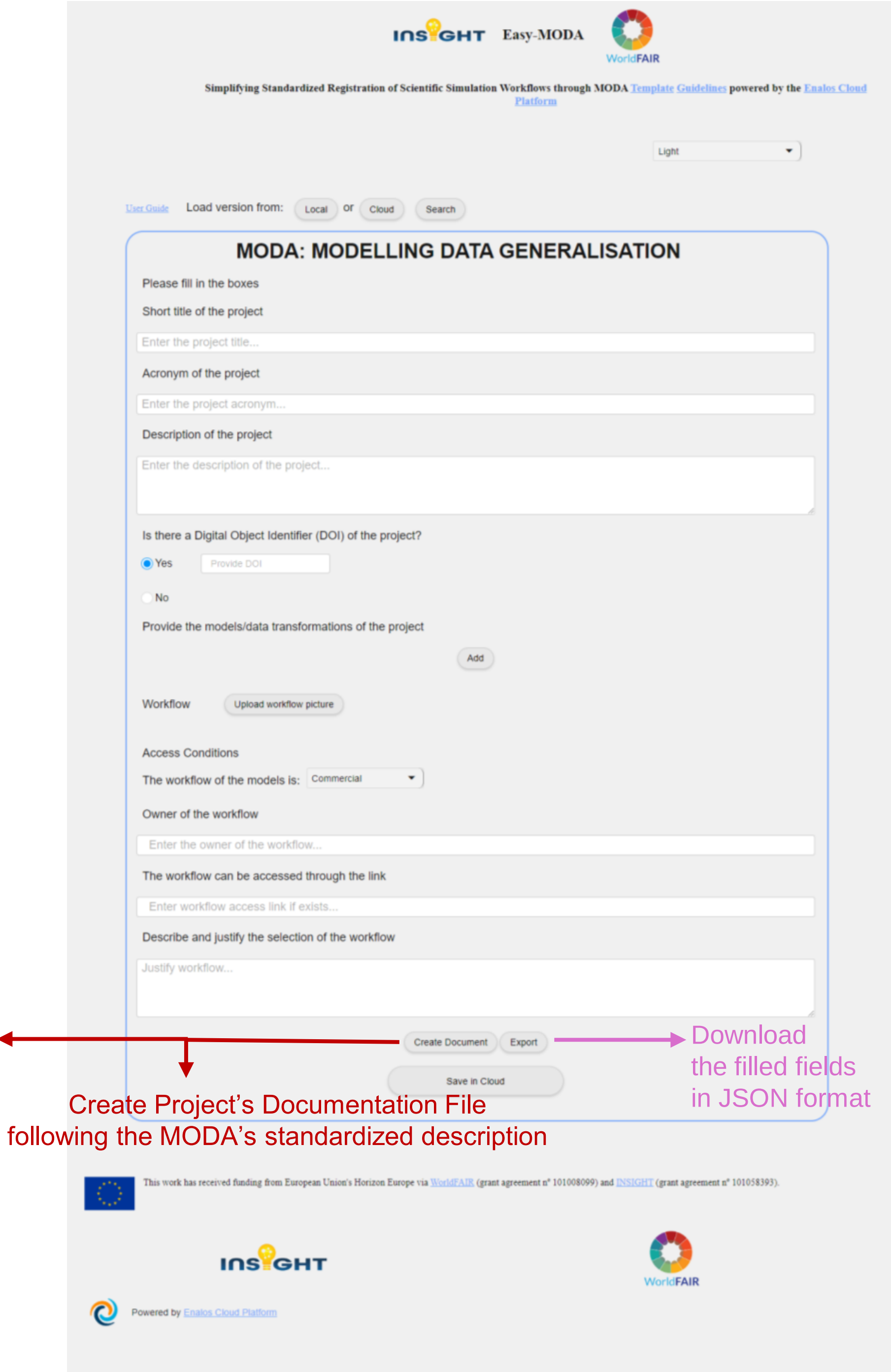


By clicking on the **"Create Document"** button (see the bottom of the right hand side of the figure), the user can get the project's information in pdf format as shown on the left of the figure) structured according to the MODA guidelines.

The user can also download a JSON file to save the information of the model(s) in a format compatible with Easy-MODA in order to upload it later.

For any further modification of the document, the user can convert the pdf file to a doc format and manually change it.

By clicking on the **"Cloud"** button (see the Figure on the right), the user can upload a user case from the cloud by inserting its 5-digit serial key .

By clicking on the **"Search"** button (see the Figure on the right), the user can search a user case from the cloud that satisfies specific criteria.

The list of the use cases of the cloud is mentioned at the bottom of the pop-up window. The user can search the list of the available use cases and load them by clicking on them. The first five digits of each model in the list are its serial key.

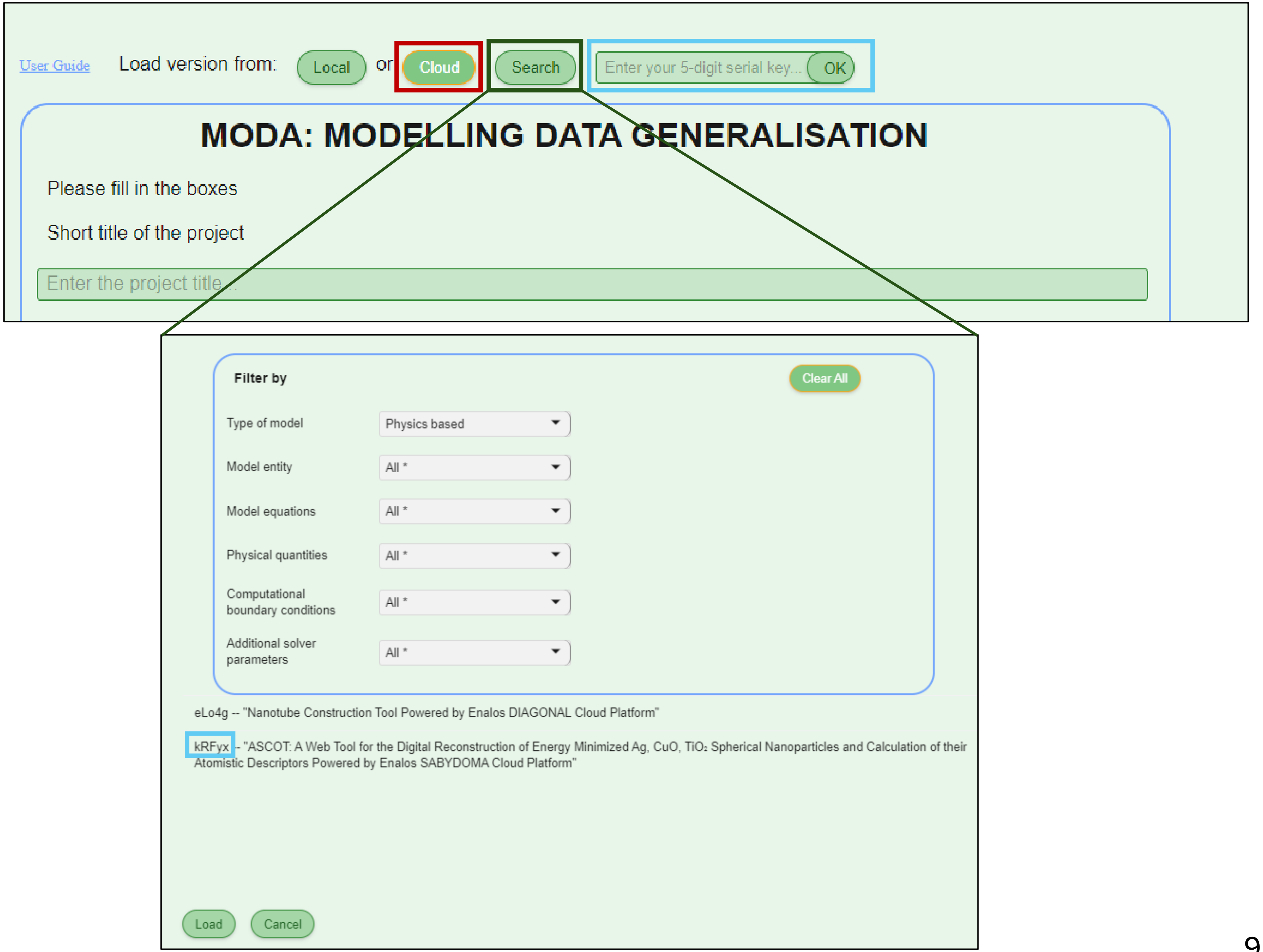

By clicking on the **“Cloud”** button (see the Figure on the right), the user can upload a user case from the cloud by inserting its 5-digit serial key .

By clicking on the **“Search”** button (see the Figure on the right), the user can search a user case from the cloud that satisfies specific criteria.

The list of the use cases of the cloud is mentioned at the bottom of the pop-up window. The user can search the list of the available use cases and load them by clicking on them. The first five digits of each model in the list are its serial key.

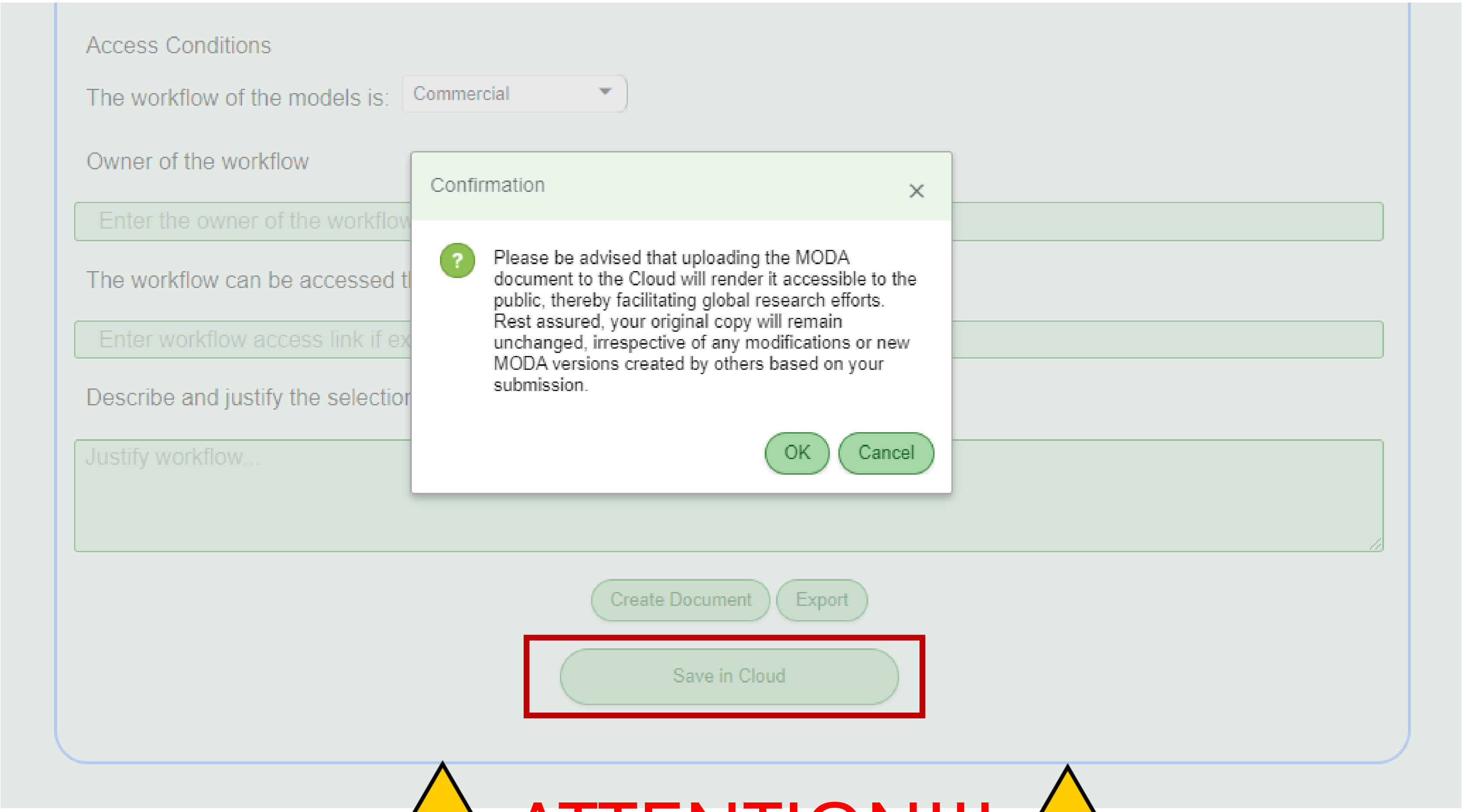


ATTENTION!!!

By clicking on the **“Save in Cloud”** button (see the Figure above), the user can upload the use case MODA documentation in the Cloud where it will remain in cloud permanently. A warning message appears to confirm that the user agrees with the uploading of the MODA document in the cloud.

https://emmc.eu/moda/

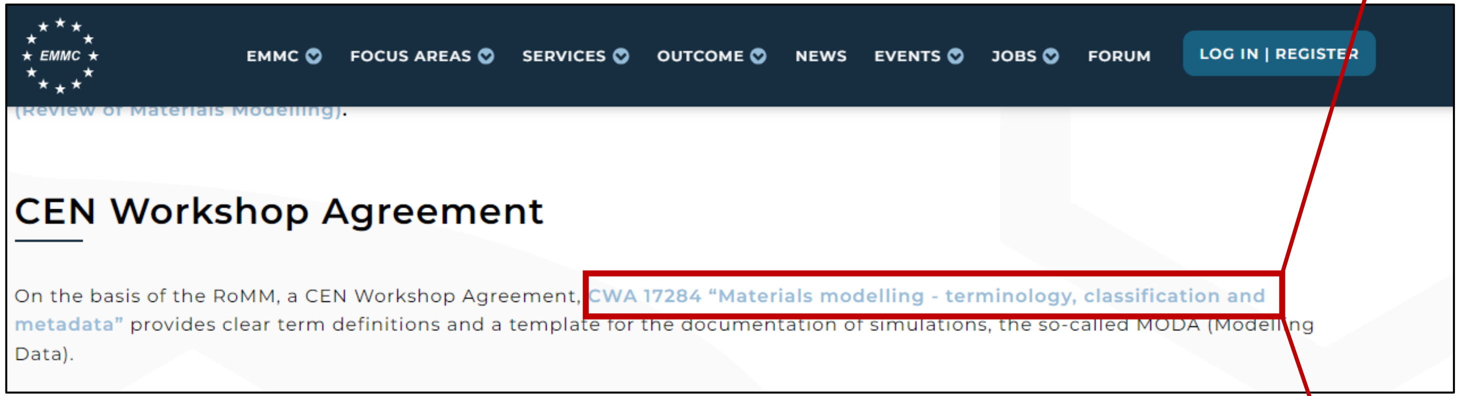


Easy-MODA complies with the CEN Workshop Agreement (CWA 17284 "Materials modelling - terminology, classification and metadata") which can be found in the EMMC webpage.

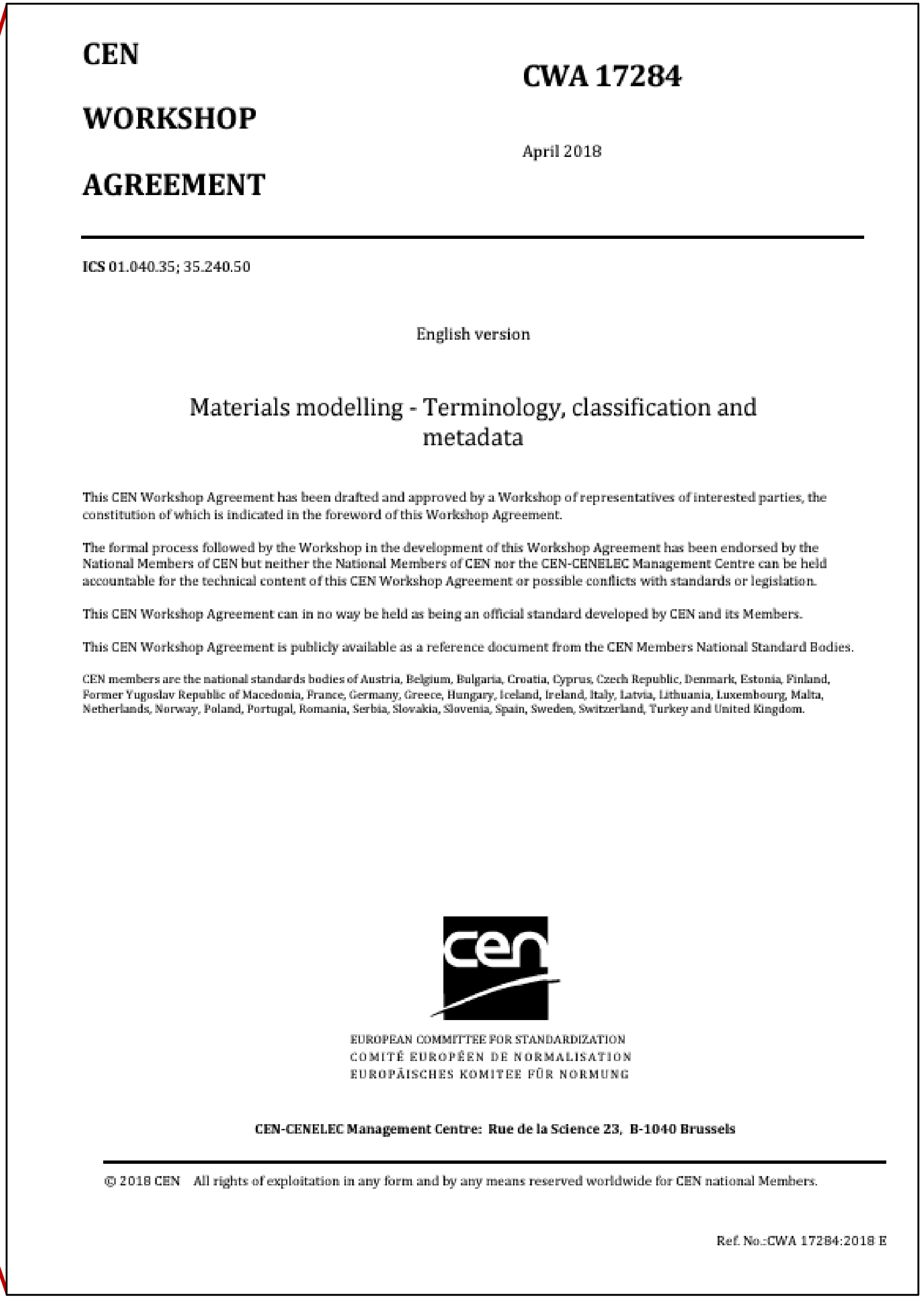

**CEN WORKSHOP AGREEMENT**

**CWA 17284**

April 2018

**ICS** 01.040.35; 35.240.50

English version

Materials modelling - Terminology, classification and metadata

This CEN Workshop Agreement has been drafted and approved by a Workshop of representatives of interested parties, the constitution of which is indicated in the foreword of this Workshop Agreement.

The formal process followed by the Workshop in the development of this Workshop Agreement has been endorsed by the National Members of CEN but neither the National Members of CEN nor the CEN-CENELEC Management Centre can be held accountable for the technical content of this CEN Workshop Agreement or possible conflicts with standards or legislation.

This CEN Workshop Agreement can in no way be held as being an official standard developed by CEN and its Members.

This CEN Workshop Agreement is publicly available as a reference document from the CEN Members National Standard Bodies.

CEN members are the national standards bodies of Austria, Belgium, Bulgaria, Croatia, Cyprus, Czech Republic, Denmark, Estonia, Finland, Former Yugoslav Republic of Macedonia, France, Germany, Greece, Hungary, Iceland, Ireland, Italy, Latvia, Lithuania, Luxembourg, Malta, Netherlands, Norway, Poland, Portugal, Romania, Serbia, Slovakia, Slovenia, Spain, Sweden, Switzerland, Turkey and United Kingdom.

EUROPEAN COMMITTEE FOR STANDARDIZATION
COMITÉ EUROPÉEN DE NORMALISATION
EUROPÄISCHES KOMITEE FÜR NORMUNG

**CEN-CENELEC Management Centre: Rue de la Science 23, B-1040 Brussels**

© 2018 CEN All rights of exploitation in any form and by any means reserved worldwide for CEN national Members.

Ref. No.:CWA 17284:2018 E
